# An agent-based framework to study forced migration: A case study of Ukraine

**DOI:** 10.1093/pnasnexus/pgae080

**Published:** 2024-03-19

**Authors:** Zakaria Mehrab, Logan Stundal, Srinivasan Venkatramanan, Samarth Swarup, Bryan Lewis, Henning S Mortveit, Christopher L Barrett, Abhishek Pandey, Chad R Wells, Alison P Galvani, Burton H Singer, David Leblang, Rita R Colwell, Madhav V Marathe

**Affiliations:** Biocomplexity Institute & Initiative, University of Virginia, Charlottesville, VA 22904, USA; Department of Computer Science, University of Virginia, Charlottesville, VA 22904, USA; Biocomplexity Institute & Initiative, University of Virginia, Charlottesville, VA 22904, USA; Department of Political Science, University of Virginia, Charlottesville, VA 22904, USA; Biocomplexity Institute & Initiative, University of Virginia, Charlottesville, VA 22904, USA; Biocomplexity Institute & Initiative, University of Virginia, Charlottesville, VA 22904, USA; Biocomplexity Institute & Initiative, University of Virginia, Charlottesville, VA 22904, USA; Biocomplexity Institute & Initiative, University of Virginia, Charlottesville, VA 22904, USA; Department of Systems and Information Engineering, University of Virginia, Charlottesville, VA 22904, USA; Biocomplexity Institute & Initiative, University of Virginia, Charlottesville, VA 22904, USA; Department of Computer Science, University of Virginia, Charlottesville, VA 22904, USA; Center for Infectious Disease Modeling and Analysis, Yale School of Public Health, New Haven, CT 06520, USA; Center for Infectious Disease Modeling and Analysis, Yale School of Public Health, New Haven, CT 06520, USA; Center for Infectious Disease Modeling and Analysis, Yale School of Public Health, New Haven, CT 06520, USA; Emerging Pathogens Institute, University of Florida, Gainesville, FL 32610, USA; Department of Political Science, University of Virginia, Charlottesville, VA 22904, USA; Center for Bioinformatics and Computational Biology, University of Maryland, College Park, MD 20742, USA; Biocomplexity Institute & Initiative, University of Virginia, Charlottesville, VA 22904, USA; Department of Computer Science, University of Virginia, Charlottesville, VA 22904, USA

**Keywords:** agent-based modeling, policy analysis, social theories, forced migration, Ukraine, digital twin

## Abstract

The ongoing Russian aggression against Ukraine has forced over eight million people to migrate out of Ukraine. Understanding the dynamics of forced migration is essential for policy-making and for delivering humanitarian assistance. Existing work is hindered by a reliance on observational data which is only available well after the fact. In this work, we study the efficacy of a data-driven agent-based framework motivated by social and behavioral theory in predicting outflow of migrants as a result of conflict events during the initial phase of the Ukraine war. We discuss policy use cases for the proposed framework by demonstrating how it can leverage refugee demographic details to answer pressing policy questions. We also show how to incorporate conflict forecast scenarios to predict future conflict-induced migration flows. Detailed future migration estimates across various conflict scenarios can both help to reduce policymaker uncertainty and improve allocation and staging of limited humanitarian resources in crisis settings.

**Significance Statement**

Conflict induced migration flows have received significant attention from both scholars and policymakers. However, estimating these flows in near real time has historically been impaired by data limitations and issues of computational scale. Leveraging a comprehensive data set of 46 million synthetic individuals and a novel agent-based modeling framework, we compute and validate daily refugee flows in response to conflict in Ukraine. Model fit is compared to alternative methods indicating the accuracy of results and policy utility is demonstrated through the use of counterfactual conflict forecasts. Model calibration can be achieved within a few days on typical hardware underscoring model utility during humanitarian crises.

## Introduction

Migration—the movement of people from an origin to a destination—has been studied extensively by demographers, economists, geographers, political scientists, and sociologists, all with differing perspectives on the causes and consequences of these movements (1–4). For the most part, the studies view migration as planned movement, with the prospective migrant intending to remain in the destination permanently or, at a minimum, for a substantial period of time. Globally, the United Nations reports that, prior to border restrictions associated with the COVID-19 pandemic, international migrants accounted for approximately 3.5% of the world’s population (5).

In contrast to *planned migration* (e.g. for education and employment), countries have increasingly experienced *forced migration*; migrations or internal displacements generated by a *shock* event. According to the United Nations High Commission for Refugees, at the end of 2019, there were almost 80 million forced migrants globally (5). A recent update by UNHCR estimates that this number has climbed to 110 million as of June 2023 (5). Forced migration differs from planned migration in that those who leave do so as a result of a conflict or a natural disaster and generally intend to return home when it is safe to do so. Understanding the dynamics of forced migration is essential for policymakers; provision of public assistance and the maintenance of civic order in countries hosting these refugees are jeopardized as a result of the abrupt nature of forced migration (6).

The 2022 Russian invasion of Ukraine represents one such shock event which generated a large-scale forced migration of Ukrainians largely into Europe. As of May 2023, 8.3 million Ukrainians fled to Europe while another 5.4 million remained internally displaced in Ukraine. Humanitarian assessments indicate that 10.2 million Ukrainians need humanitarian assistance (7). In order to meet these needs, it is imperative that policymakers in host countries as well as humanitarian organizations leverage all available information to make resource and logistic staging decisions to aid inbound refugees requiring assistance (8). However, the sudden nature of forced migration events makes such staging decisions nontrivial and, in the past, reactionary responses have in some cases failed to meet the needs of large refugee flows (9). Therefore, a framework to estimate forced migration based on underlying geopolitical or environmental shocks is critical.

Traditional models existing for migration are mainly used in the context for *planned migration* and they are functional in nature. For example, the Gravity model (10) estimates migration from a source location to a destination location primarily based on distance, and the Radiation model (11) does so through the population of the two locations and the population in the intervening locations. However, forced migrations primarily occur due to external events (e.g. a conflict event or a disaster) (12), making such models less suitable for this purpose, since these models do not provide a straightforward way to incorporate the effect of such events. Political and social scientists have developed multiple theoretical accounts of human behavior and decision-making during migration, and the extent to which these theories can help explain *planned migration* has been studied extensively. Popular theories include but are not limited to, utility maximization (1), theory of planned behavior (13), and herd effect (14). However, these theories have not been studied in *forced migration*, though it has been suggested that models driven by social theories can essentially be useful in situations with no or inadequate data (15) prevalent during *forced migration*.

To address these limitations, we propose ABSCIM (Agent-based simulator for conflict-induced migration), a data-driven, theory-guided, agent-based framework to model forced migration. Necessity of the *data-driven* part of the framework arises from the fact that uncertainty and noise are always lingering issues associated with data regarding forced migration events (6, 16) and uncertainty remains with respect to the kind of data essential to drive the framework. We take a step towards filling this gap by combining real-world conflict information with digital twins (17, 18) in the form of a synthetic population. The necessity of a *theory-guided* model emerges because a framework using social theories provides a strong foundation that will be accepted by policymakers and has greater potential to accurately model human behavior than alternatives (15). To address this, we embed necessary social rules to the decision process of the synthetic individuals when they interact with the real-world conflict events. Furthermore, because migration is ultimately a macro-level phenomenon resulting from the aggregation of micro-level individual decisions (16), an agent-based model (ABM) is suitable for mimicking this bottom-up behavior. By embedding the decision-making process of each synthetic individual as rules derived from social theories and having them react to real-world events under the hood of an ABM, we can obtain meaningful results. Thus, it provides the opportunity to relate to observed behavior and flexibility to analyze different aspects of forced migration. Given that agents are micro-level entities, an ABM can generate both high-resolution and low-resolution information, underscoring its usefulness in generating data nontrivial to obtain in real-world scenarios. Moreover, it is possible to define behavioral patterns of agents of various demographic groups separately, supporting fairness and equity. Finally, policymakers seek the ability to perform various counterfactual analysis to help them understand various uncertainties and plan accordingly. Since ABM allows decomposition of parts and factors, selection of tangible targets and incorporation of theoretically guided decision-making process along those targets will help policymakers to achieve this by providing understandable “levers” to change in the model.

This study investigates the following questions in the context of forced migration from Ukraine. First, how to integrate social theory into an ABM that can compute migrant outflows from a conflict-induced region? Second, how effectively can an ABM generate estimates at fine temporal, spatial, and demographic resolutions? Last but not least, what are the policy implications of such a model? ABSCIM uses the *Theory of Planned Behavior* as a foundation for agent decision-making. Combining the conflict events of real-world data with the decision-making process of the digital twins of the real population, ABSCIM can generate meaningful results. Temporally, it generates *daily* migrant outflows from Ukraine. Upon comparing with available border-crossing reports, we obtain a Pearson Correlation Coefficient (PCC) of 0.96, suggesting ABSCIM is able to capture the temporal trend very accurately. Spatially, the ABM can disaggregate conflict-induced displacement at *any* spatial scale such as Admin-2 level Raions within Ukraine. Finally, since we model individual agents by using a synthetic population developed from publicly available data (19, 20), migration estimates can be aggregated based on demographic characteristics including age and gender. We demonstrate policy relevance of the model in two ways. First, we leverage the ABM’s data-rich outputs to explore the prevalence of wartime sexual assault—an understudied problem the study of which is impaired by data limitations our model helps to address. Second, we develop two counterfactual conflict forecasts to demonstrate how the model can integrate into policymaker analyses. Both policy applications underscore how the proposed ABM framework can generate new high-resolution theoretically grounded data that can reduce uncertainty and aid in resource staging during forced migration events.

## Related work

This section covers a subset of the most relevant literature. Appendix SA Section 1 discusses additional related work.

**Forced migration:** Davis et al. (21) estimate the number and destination of migrants due to sea level rise by incorporating the number of migrants from potential destinations in the radiation model and the additional resource incurring as a result of such migration. However, the model calculates the number of estimated migrants from destinations by using a proportional model from historical data which is fairly simple. It also does not consider other behavioral dynamics (e.g. return migration, peer effects, different migration capacities of different demographics) associated with shock migration. Extending their approach, De Lellis et al. (22) account for unwillingness to migrate and return migration by introducing the entire model dynamics in the form of linear equations and incorporating one additional parameter. Additionally, these papers primarily focus on the economic factors in choosing the destination of the migrants. A very recent work by Pandey et al. (23) tries to assess the health situation of Ukrainian refugees resulting from the Russian invasion. The focus of that paper is to estimate health impacts due to the conflict; further, the computational approach in that paper differs from the one studied here. The agent-based approach outlined here naturally provides a way to incorporate individual and collective behavior to produce emergent migratory flows.

**Agent-based modeling:** Nelson et al. (24) model the displacement of Somalian shepherds under the influence of civil war and environmental factors. The model finds that access to vegetation has a correlation with the shepherds’ movement. However, the correlation with conflicts still remains unclear. Collins et al. (12) propose an agent-based model driven by utility function to understand how group movement happens during migration. Closely associated with our work, Hebert et al. (25) do not consider different demographics, are not generalized in choosing destination, and do not consider peer effect. The work by Suleimenova et al. (26) is perhaps the closest to us where they try to predict how refugees will choose among a set of camps by simulating three conflicts in Africa. However, they focus primarily on identifying destinations of the refugees, given the displaced population has been identified already. On the other hand, our model focuses on identifying this initial displaced individuals.

**Social theories for migration:** Various social theories have been explored, but mostly in the context of *planned migration*. For example, the micro-economic expected utility theory describes how individuals choose among a set of discrete choices. Biondo et al. (27) consider return migration for brain drain by considering that agents always try to maximize their income. A similar work is by Garcia-Diaz (2) where agent changes their states based on utility calculated from income and neighbors in that state. Apart from that, another popular theory is to explain the action of a respondent in response to an individual. Kniveton et al. (15) use the Theory of Planned Behavior to explore an individual’s decision to migrate under climate change. Smith et al. (28) also use the same theory to understand rainfall-induced migration. However, as mentioned earlier, these theories have no mathematical formulation and they have not been explored in the context of forced migration.

## Methods and data

### Model dynamics

The main input space of our problem encompasses a set of conflict events $C=\{c_{1},c_{2},\ldots ,c_{j},\ldots \}$, a set of person agents $A=\{a_{1},a_{2},\ldots ,a_{i},\ldots \}$, a set of household agents $H=\{h_{1},h_{2},\ldots ,h_{k},\ldots \}$ in the affected region. We also assume we know $N(h_{k})\subseteq H$, the neighbors of each household $h_{k}\in H$. Finally, we are given the mapping function η:A→H, representing the household of each individual. The two types of agents create a hierarchical agent-based structure where the decision of person agents translates to their households and the final decision to migrate is taken at the household level. We assume when a household migrates, all the associated person agents also migrate.

**Fig. 1.**
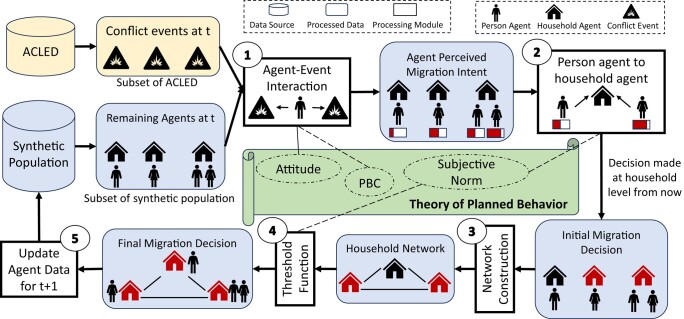
Architecture of the model. After extracting the agent data (from synthetic population) and conflict data (from ACLED) for the current timestep, (1) Each person agent interacts with conflict events in their vicinity and develops an initial perceived migration intent (Attitude and PBC), (2) Agents in the same household combine their decisions to form a unified initial migration decision at the household level (Subjective Norm), (3) A network of household is constructed (see Appendix SA for details of the construction mechanism), (4) Each household agent communicates with household agents in their neighborhood through the threshold function to ultimately decide whether they migrate or not (Subjective Norm), and (5) Migrating agents are removed from consideration for next timestep.

Our model adopts the popular *Theory of Planned Behavior* (29) approach as its underlying social theory. This theory has been used in the past in the context of planned migration (13). In its general form, the theory states that the outcome in response to risk depends on three things: (a) *Attitude* towards the risk, (b) *Perceived behavior control (PBC)* or individual perception about the ease or difficulty of performing the behavior, and (c) *Subjective norm* or belief about whether the action is reflective of the action of their peers. Since we adopt this theory, our model includes all three components, which we describe next. Figure 1 presents a holistic visualization of how the model operates.

**Attitude:** Intuitively, an individual’s attitude towards the risk can be correlated with the impact of the events associated with that risk (13). Towards that end, the observed impact of a conflict event $c_{j}$ by an agent $a_{i}\in A$ at time *t* is modeled by the following equation:


(1) $$f^{\prime }(i,j,t)=\frac{I_{j}}{\Delta_{T}(t,T_{j}{)}^{\tau }\cdot \Delta_{S}(x_{i}(t),L_{j}{)}^{\delta }},$$

which is an extension of the equation proposed in (30). Here, $I_{j}$ is the intensity (see Appendix SA for details on intensity calculation in our context), $T_{j}$ is the time, $L_{j}$ is the location of conflict event *j*, and $x_{i}(t)$ is the location of agent *i* at time *t* if they have not migrated yet. $\Delta_{S}$ and $\Delta_{T}$ are functions to compute the spatial and temporal difference between two locations and times, respectively. *δ* and *τ* are the spatial and temporal decay parameters (δ>1,τ>1).

By following that the total impact of events on a respondent is given by the sum of the individual event’s impact (30), the attitude towards risk is given by:


(2) $$f(i,t)=\sum_{c_{j}\in C(t)}\;f^{\prime }(i,j,t),$$

where $C(t)=\{c_{j}|T_{j}<t\}$ is the set of events that happened until timestep *t*. Note that, the incorporation of the term $\Delta_{T}(t,T_{j}{)}^{\tau }$ in the denominator of Eq. 1 implicitly incorporates a discounting factor on events that happened before time *t* in Eq. 2. For example, choosing $\Delta_{T}(t,T_{j})=t-T_{j}$ will result in exponential discounting.

**Perceived behavior control:** The same impact can be perceived differently by different agents. For example, older people and children are more affected by socio-political violence than other groups (31, 32). Following this, the perceived impact of agent $a_{i}$ at time *t* is given by:


(3) $$\tilde{\;f}(i,t)=\beta_{i}f(i,t)+\theta \;\;\tilde{\;f}(i,t-1).$$

Here, $\beta_{i}$ is the risk-proneness (details in Appendix SA) and *θ* (0≤θ≤1) as the memory retention of agent $a_{i}$. The first component is dependent upon the inherent attributes of the agent and controls how much the current impact is perceived by a person agent. The second component controls how much of the past impact is remembered by the agent. For simplicity, we consider that this parameter does not vary across agents. Next, this perceived impact is converted to an initial probability to migrate through a logistic function:


(4) $$p^{\prime }(i,t)=\frac{1}{1+Qe^{-v\;\;\tilde{\;f}(i,t)}}.$$

Here, *v* is the growth rate, and *Q* controls the probability of migration of an agent given their perception of risk is 0.

**Subjective norm:** Although the previous constructs were formed at the person-agent level, this construct is formed at the household agent level. We incorporate the notion of peer effect to capture this construct. Peer effect refers to how the behavior of others affects the behavior of an individual and it has been studied in the past in the context of migration (33, 34). Using the inverse image of agent-to-household mapping *η* and the previously computed migration probability of person agents, we account for *intra-household* peer effect, by defining the initial migration probability of each household agent $h_{k}$, which is collectively formed through the average probability of the person agents of that household as follows:


(5) $$p(k,t)=\frac{1}{\left|\eta^{-1}(h_{k})\right|}\sum_{a_{i}\in \eta^{-1}(h_{k})}\;p^{\prime }(i,t).$$

Here, the summation term accounts for the migration probability of all agents of the household $h_{k}$, and it is normalized by the cardinality of $\eta^{-1}(h_{k})$, which represents the number of person agents living in $h_{k}$.

Based on this collective probability, the decision to migrate is sampled from a Bernoulli distribution as follows.


(6) $$m^{\prime }(k,t)=\left\{ \begin{array}{cc} 1 & \text{with probability}\;p(k,t)\\ 0 & \text{with probability}\;1-p(k,t). \end{array}\right.$$

Finally, to account for *inter-household* peer effect, we employ a threshold-based migration function, motivated by threshold function, a standard practice used in the context for peer effect ((35, 36)), and update the migration probability of each household by looking at the neighboring households. Formally, given that $N(h_{k})$ is the set of households in the neighborhood of $h_{k}$, the adjusted migration decision for household $h_{k}$ at time *t* is given by:


(7) $$m(k,t)=\left\{ \begin{array}{cc} 1 & \text{If}\;\sum_{h_{u}\in N(h_{k})}m^{\prime }(u,t)>I_{hi}\;\\ 0 & \text{If}\;\sum_{h_{u}\in N(h_{k})}m^{\prime }(u,t)<I_{lo}\;\\ m^{\prime }(k,t) & \text{otherwise}. \end{array}\right.$$

Once migration decision is taken at the household level, all the associated person agents migrate based on the assumption we made and they do not participate in the perception-action loop for subsequent timesteps. For a summary of the notations and the parameters of the model, please refer to Appendix SA. Note that, this model accounts for only the initial displacement of the population due to conflict events. Once an individual is displaced, they can make subsequent trips to other places or even return home. Since understanding the destination is out of the scope of this model, our model is best suited for estimating the initial displacement during the shock period of a conflict scenario, when information is the most scarce.

### Calibration

Calibration involves choosing suitable values for the parameters used in an ABM so that they produce realistic outputs. Since ABM can generate a multitude of outputs, calibration is performed after selecting some outputs for which observable data is available. For our context, we calibrate our model against the daily border crossing data (described in the next section) with respect to the daily refugee estimations generated by the ABM. We employ a calibration technique based on the coordinate descent optimization algorithm. The details of the calibration technique are outlined in Appendix SA.

While designed to be generalized for other conflict settings, a limitation of our model is that depending on the nature of the conflicts and the nature of the agents in the conflict-induced region, the necessity of recalibration may arise. Here, we discuss briefly parameters that may require recalibration in different conflict settings and parameters that can be thought of as global parameters, even across different conflict scenarios.

**Decay parameters:** The temporal decay (*τ*), the spatial decay (*δ*) and the memory decay (*θ*) parameters can be generalized across different conflict settings. In fact, literature exists with recommendations for the memory decay parameter, which we use for the study of Ukraine. Therefore, once calibrated for one conflict setting, these parameters do not require recalibration.

**Migration control parameters:** The two migration control parameters used in Eq. 4 may require recalibration in different conflict settings. For example, the no-risk migration (*Q*) parameter controls the probability of migration of an agent even if they have no perception of risk. A low value of *Q* will be associated with a high probability of migration even without the perception of a risk. This can happen in conflict settings where the perpetrator of violence resides inside the territory or the target agents belong to a minority class. Similarly, the growth rate parameter (*v*) controls the rate of increase in agents’ intention to migrate with the increase in risk perception. A higher value of *v* will make agents prone to migration even for low-risk perception. However, there is certainly a threshold for *v* beyond which the intention will look practically the same since it is in probabilistic space. While this is not within the scope of our study, sensitivity analysis of the model is worthy of efforts to understand these dynamics. Note that, there were two additional parameters added (bias scale *b* and conflict scale *w*, details in Appendix SA) for scaling the perceived impact of the events. However, the design of our model implicitly implies a correlation between *v* and these scaling parameters as v=zbw, where *z* is a hidden parameter. Moreover, since b,w∈[0,1], they do not change the initial range of *v*. Therefore, calibration of *v* indirectly implies scaling of these two parameters and therefore recalibration of *v* is sufficient in the context of the scaling parameters as well.

**Threshold parameters:** The appropriate value of the two threshold parameters in Eq. 7, $I_{hi}$ and $I_{lo}$, will depend on the structure of the neighborhood of the agents. However, since obtaining information at such granularity is not possible in real-life scenarios, these need to be calibrated empirically.

To demonstrate the generalizability of the modeling framework to other conflict contexts as well as the feasibility of calibration in other conflict contexts see Discussion in the Results section and more detailed discussion in Appendix SA.

### Data description

**Agent data:** The concept of digital twin describes the one-to-one correspondence between synthetic information and its real counterpart, and it has received considerable attention among researchers of social simulations (37). Synthetic information about a population, if statistically similar, can be used to study realistic behavioral patterns. Motivated by this, we use the synthetic population data constructed using Census data and other sources by the Biocomplexity Institute (20). It contains (i) the demographic attributes of each individual (e.g. age, gender) and (ii) their partition into households with household attributes (e.g. household size and location). The synthetic population is constructed so that they closely resemble their real counterparts. Given that it has a statistically similar representation to the actual demographic distribution, this data provides us with a reasonable microscopic ability for simulating the actual scenario. The synthetic population data for Ukraine contains ∼21.2 million males and ∼24.7 million female individuals, spanning ∼19 million households. For simplicity, we consider an agent’s location to be the same as their household’s.

**Conflict data:** We obtained conflict data from the Armed Conflict Location & Event Data Project ((38)). ACLED is a widely used dataset that provides detailed information on political violence and protests across Africa, the Middle East, and South Asia. It has data available from 1997 up to the present, with daily updates. It captures a wide range of events, including riots, protests, battles, explosions/remote violence, violence against civilians, and strategic developments (such as the signing of peace agreements). It also provides information about the location, time of the event, the actors involved, and the number of fatalities and injuries.

In our work, we extract the conflict events pertaining to Ukraine from 2022 March 1 to 2022 May 15, due to this being the shock period of the war, a phase worthy of exploration and study. A total of 5,645 events are recorded across Ukraine in this dataset. Among them, we focus on three types of events that are likely to cause damage to infrastructures as well as human lives—explosions, battles, and violence against civilians which include events where conflict belligerents intentionally target or harm civilians or noncombatants.

**Border crossing data:** Mainly used for calibration purposes, we utilize the border crossing data from Humanitarian Data Exchange (HDX) (39) collected between 2022 March 1 to 2022 May 15. We note that there is a paucity of time series data on people leaving home from Ukraine during the initial period of the war. The border crossing data are the next best thing that was available. However, there are certain idiosyncrasies associated with such data. For example, the agencies who track such movement have no way to track each person’s movement (40). However, it is still valuable data that will provide some context to how reliable our model is since this is data that is at daily temporal resolution and something that we can use to validate our model. See Appendix SA for additional details on border crossing validation as well as agent decisions to flee as refugees or internally displaced.

## Results

In this section, we primarily highlight the ability of our model, which we refer to as ABSCIM (Agent-based simulator for conflict-induced migration), to produce fine-grained spatiotemporal data in the context of the Russian invasion of Ukraine. We calibrated the model in less than four days in an HPC cluster with limited available concurrent memory (384 GB) and available concurrent nodes (40). In Appendix SA, we also describe ABSCIM estimation in the context of a past conflict of Northern Mali. The number of reported border crossings from Ukraine during our study period from March 1 to May 6 is ∼5.16 million. Our model, without taking any historical data as input, generates an estimated median of 5.64 million refugees within the same time period. In addition to these aggregate total refugee estimates, by recording the date, location, and demographic characteristics of agents at each timestep during simulation, ABSCIM can also produce granular estimates at fine temporal, spatial, and demographic resolutions.

**Fig. 2.**
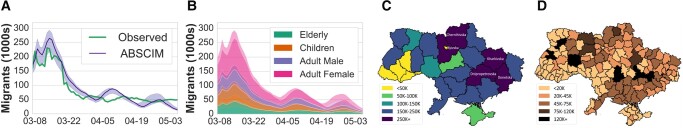
Spatial, temporal, and demographic dimensions of ABSCIM estimation capability. a) shows the daily estimation of total individuals crossing borders, compared against reported border crossings for validation. Both data are displayed with a 7-day moving average applied to reduce noise associated with the reported data (unsmoothed observed counts appear in Appendix SA). b) presents stack plots disaggregating refugee totals into four different demographic groups. Validation is not possible due to the absence of observed data. However, we present validation against some early qualitative reports indicating aggregated statistics, presented in Appendix SB. c) shows the cumulative total median outflow estimation at Oblast level. A high density of estimated migrants can be observed across the Eastern regions, where early conflict events took place. *Dnipropetrovska*, *Donetska*, *Kharkivska*, and *Kyivska* oblasts were reported to be among the top five oblasts of origin for the refugees during the early period of the war (UNHCR), which are among the top five oblasts to have the highest refugee estimates by the ABM. d) disaggregates the outflow at Raion level. See Appendix SA for visualizing the ratio of refugee agents with respect to the total population. (a) Total. (b) Demographic. (c) Oblast and (d) Raion.

**Daily estimation:** Figure 2a presents two notable features highlighting the overall performance of ABSCIM to capture the underlying dynamics of conflict-induced migration. First, when compared against reported border crossing data, ABSCIM performs very well capturing the shape of the overall outflow, particularly the surge in early March as well as a later relatively smaller surge in early April. The Pearson correlation coefficient (PCC) value between the reported border crossing data and ABM estimates is 0.96, which quantitatively tells us that ABSCIM does a very good job of capturing the overall trend of the daily border crossing from Ukraine. Impressively, ABSCIM captures waves of migration using only the synthetic population data and conflict event inputs. Second, beyond the overall shape of the refugee flows, the total daily number of refugees estimated from the model closely maps onto the numbers reported in border crossing data. In order to understand the goodness of fit of ABSCIM, we compare its performance against a vanilla regression method trained to predict the daily refugee outflow and find that ABSCIM is better in both estimating the trend and the scale of the outflow. Please refer to the Appendix SA for details of the analysis. Overall, the credible interval very closely overlaps with the reported border crossing data for most days in the analysis period.

**Daily demographic trends:** Figure 2b disaggregates the total daily refugee estimates from ABSCIM presented in Fig. 2a breaking them down into broad demographic age categories (elderly, adults, and children) as well as gender (male, female). As an additional point of validation, we also compare estimated demographic distributions with the reports for the first four rounds conducted by the International Organization of Migration (IOM), the details of which are present in Appendix SB. The capability of generating information at such fine demographic resolution is unique to the ABM approach that leverages a synthetic population of Ukraine and underscores the policy relevance of the framework here. By generating demographically detailed *daily* estimates of conflict refugees fleeing Ukraine, more tailored policy and humanitarian responses can be crafted. We explore this potential further in Appendix SA where we show the estimation broken down by various age groups. Such breakdown would be relevant for healthcare professionals seeking to respond to the particularized needs of refugees across medically relevant age cohorts. Of course, these categories could be further disaggregated by gender as well. Such detailed data can help to identify a number of important features of the refugee population, for example, whether the composition of refugees is changing over time, indicating a need for flexible policy responses to accommodate changing refugee needs as a conflict unfolds. Beyond these age and demographic characteristics, the geographic origin of these refugees is also recorded for each time step in the model, offering additional opportunities to understand refugee conditions (e.g. distance traveled) as well as areas of Ukraine requiring refugee resettlement assistance after the end of hostilities.

**Spatial-subnational analysis:** Similar to the preceding analysis, ABSCIM can also estimate migrants at a fine level of spatiality (Fig. 2c and d). Spatially explicit estimates of the total number of civilians displaced by armed conflict at the Oblast or Raion level within Ukraine over the course of the conflict are difficult to obtain. This data sparsity issue is more pronounced in regions with greater levels of violence in which accurate data collection and reporting are compromised owing to the risks that humanitarians and international organization personnel face in such environments. However, data of this nature are crucial for understanding which areas of a crisis space have suffered the greatest out-migration and are, therefore, most likely in need of reconstruction and resettlement support, a topic revisited in the conclusion. The granular nature of the temporal and spatial estimates that emerge from the ABM underscore both the strength of this approach but also the policy relevance providing displacement estimates at various levels of aggregation relevant to policy analysts. Furthermore, the spatial maps shown here provide only a cumulative snapshot of displacement outflows over all the days in our study period. These displacement maps can be also generated for each day of the conflict thereby helping to generate additional necessary insights into the locations of origin for newly arriving refugees.

The cumulative displacement estimates highlight the level of spatial granularity that emerges from the model with the results strongly suggesting that those areas most heavily impacted by violence also represent the origins of the greatest number of displaced Ukrainians. Considering Fig. 2d, estimates of displaced agents have been aggregated to the Raion (administrative-2) level. The results demonstrate that ABSCIM appropriately identifies the heaviest migration from Raions which, during this initial stage of the war, suffered heavy ground-force combat action or more remote violence such as by enduring artillery strikes and cruise-missile attacks. Indeed, satellite analysis of cities such as Lyman, Severodonetsk, and Lysychansk, located in Raions with some of the largest modeled migration estimates, suggest that between 20 and 30% of buildings and infrastructure in these locations had suffered damage as a result of conflict during the initial stage of the war analyzed here (41). These spatial results complement the daily and demographic analysis to illustrate the potential of the ABM to generate policy-relevant insights in near-real time as a crisis such as the Ukraine conflict unfolds.

## Policy implications

Policymakers frequently must respond to crisis situations while confronting uncertainty and information asymmetries that impair their ability to effectively implement policy and craft tailored solutions in new situations (42). The mass influx of refugees at the onset of the Ukraine conflict represents such a crisis situation. During the first 30 days of combat operations in Ukraine starting 2022 February 24 and ending March 26 ABSCIM suggests that 4.27 million Ukrainians had fled their country with the vast majority fleeing into neighboring Europe (4.45 million reported in border crossing data). This represents the largest migration in Europe since World War II. The spatial, temporal, and demographic granularity of migration flows computed by ABSCIM can provide policy makers with critical data during a crisis which we explore here through analysis of the model’s demographic data outputs and through counterfactual conflict analysis.

The extent of the Ukrainian refugee crisis demanded swift policy responses despite many uncertainties associated with the scale, composition, and, therefore, particular needs of the inbound refugee population. For example, EU officials at various levels acted at the outset of the crisis by, for the first time, activating the Temporary Protection Directive granting Ukrainian refugees temporary residency, employment rights, and access to social services among other protections (43). By the beginning of March the EU Commission implemented the Cohesion’s Action for Refugees in Europe act to facilitate a more fluid transfer of funding to meet growing refugee resource needs such as for trauma counseling, food and housing, or job support (44). However, by the end of March the Commission had also recognized that data limitations had impaired the ability of member-states and NGOs to effectively respond to the crisis and, therefore, proposed a 10-Point coordination plan. This plan, among other directives, called for member-state contingency planning and information sharing on inbound Ukrainian refugees to better match refugee needs with excess humanitarian capacity (45). Indeed, the initial efforts to support the first wave of Ukrainian refugees were largely ad hoc endeavors by local NGOs and municipal governments or private citizens, bearing anticipation uncertainty as the newly started conflict unfolded (46).

The scale of the refugee crisis, especially during the initial weeks of combat, increased the chance that data limitations would impair the efficiency of policy and humanitarian response. We know that humanitarian organizations and governments alike have miscalculated with respect to refugee flows in the past. For example, in the lead-up to the 2003 Iraq invasion, uncertainty about the effect of violence on civilian displacement caused aid organizations to prepare displacement camps for an anticipated wave of fleeing Iraqi refugees which never materialized (47). A modeling framework like ours, which integrates conflict data to provide estimates on the composition of refugees or may be used to plan for varied scenarios can serve as a tool to help inform humanitarians responding to refugee crisis situations. While the outputs of ABSCIM have several potential policy applications, here we illustrate two which underscore the utility of the model as a tool that can help reduce uncertainty associated with refugees fleeing violence. We first examine detailed demographic data on gender and age generated by ABSCIM to evaluate the prevalence of wartime sexual assault—a frequently overlooked form of violence in armed conflict and a widely reported form of abuse committed in the ongoing war in Ukraine. Following this, we produce and analyze three scenario-based conflict forecasts to predict anticipated refugee flows under varied future conditions.

**Demographic analysis:** Among the many alarming elements of Russia’s invasion of Ukraine is widespread reporting of sexual assault and rape of Ukrainian women by Russian armed forces (48). Some at the UN have suggested that the scale of abuse reflects deliberate tactical choices on the part of the Russian military, possibly to dehumanize and incite fear among the civilian population (49, 50). Both the United Nations and Human Rights Watch have independently documented several cases of reported sexual assaults occurring during the first months of the war with victims ranging between 4 and 80 years old (51, 52). However, the extent of abuse and therefore need for support among refugee wartime sexual assault victims is difficult to quantify (53). This is not only partly due to issues of data collection on refugee needs (46) but also to underreporting associated with victim feelings of stigmatization or shame, or feelings of helplessness attributable to real or perceived lack of services available for refugee sexual assault victims (54). Recent analysis of Ukraine wartime sexual violence suggests that limited data availability, especially data on refugee and IDP flows broken down by gender and age cohorts, has significantly impaired service provision and needs assessment for adequately responding to victims of gender-based violence among Ukrainian refugees (55).

Since ABSCIM can provide daily refugee and IDP flow estimates disaggregated by gender, age, and location of origin, it can serve as a policy tool to fill data gaps impairing decision-making. Numerous health ramifications of sexual assault including increased risk for HIV (56) as well as psychological trauma or increased risk of suicidal ideation (57, 58) have motivated efforts to quantify the prevalence of sexual assault among refugees fleeing armed conflict. Despite these efforts, significant uncertainty and regional heterogeneity exist in these estimates. A recent meta-analysis of wartime sexual assault suggests that on average 21%, or 1 in 5 women fleeing armed conflict experience some form of sexual violence (59); however, the value may range from as low as 4.4% to as high as 43.5% depending on the conflict.

Using ABSCIM estimates of daily adult female refugees (17–50 years) (refer to Appendix SA for figures) here we compute the potential number of sexual assaults to have occurred in Ukraine during the initial phase of the war. Given the uncertainty of sexual assault prevalence here we conservatively employ lower bound estimates reported in (59) across all conflicts (14.9%) and using estimates of sexual assault for two conflicts in Europe: (Bosnia 37.6%, Kosovo 3.4%). Using these sexual assault estimates and detailed demographic and spatial data computed by ABSCIM we estimate the extent of possible sexual violence in Ukraine during the start of that conflict (see Appendix SB for additional analysis accounting for uncertainty of sexual assault prevalence and the value of this policy application of ABSCIM). Our analysis suggests approximately 1.02 million women fled from regions with active Russian military presence in the week leading up to their decision to flee. These civilians faced the greatest risk of potential sexual assault. Based on best estimates of the prevalence of wartime sexual violence and the total flow of at-risk civilians computed by ABSCIM, this suggests that as many as 152 k [35 k, 385 k] may have suffered some form of sexual violence during the initial phase of the war. Appendix SB provides additional details describing these estimates and their uncertainty.

Two things are worth noting regarding this baseline estimate. First, given the prevalence of reported assaults for victims fleeing Russian-held regions, these figures may represent an understatement of the total scale for this form of violence. Second, heterogeneity among refugees would also indicate that the problem may be more pronounced for later migrants. Ukrainians in the initial wave tended to have greater resources and familial connections in Europe leading to their early migration (46) and past political violence work strongly suggests that later migrants lack similar financial or social resources (60) which may indicate a need for greater financial support in this area as the conflict progresses. Since refugees fleeing conflict later were more likely to have encountered Russian military forces, the likelihood of their having suffered some form of sexual violence is higher. Nonetheless, these estimates demonstrate how demographically detailed data computed by ABSCIM can be used to compliment analyses by reproductive health experts and trauma specialists. Furthermore, the risks of sexual violence that women face while fleeing armed conflict do not end once leaving the conflict space and include elevated risks of human trafficking once arriving in neighboring countries (54, 61). Therefore, empirically informed estimates provide a timely sense of both the scale and resource need for a specific policy problem and therefore demonstrate how the usefulness of ABSCIM. Finally, (to our knowledge) no other estimates of the potential scale of wartime sexual assault in Ukraine yet exist and thus these estimates from ABSCIM are a useful complement to ongoing policy work.

**Forecast scenario analysis:** Conflict forecasting has long served as a goal for empirically oriented political violence scholars as a mechanism to assist policymakers in responding to crisis situations (62–64). The underlying assumption is that having empirically informed estimates of future conflict, governments and NGOs alike could, redeploy personnel to reduce risk of harm or prepare resources for refugee flows. However, absent a model of the relationship between conflict and civilian displacement, conflict forecasts would be of more limited use to policymakers most interested in responding to the outcomes of conflict rather than the conflict itself. By integrating a conflict forecast directly into ABSCIM, here we overcome these limitations and produce empirically informed estimates of anticipated future refugee flows that can directly inform policymaker decision-making. Towards that end, we utilize a Log-Cox Gaussian point process model with a spatial mesh (see details in Appendix SB) to create three distinct conflict scenarios that policymakers would have considered as plausible outcomes in May 2022 and analyze the results, to illustrate the efficacy of ABSCIM for such counterfactual analysis which policymakers and conflict analysts could potentially incorporate into their analysis workflows. These scenarios begin from 2022 April 21; by when ABSCIM has approximately estimated displacement (either IDP or refugee) of 8.75 million household agents (23.59 million person agents) out of 18.99 million household agents (45.81 million person agents). This approximates 51.5% of the Ukrainian population and the remaining population represents a significant portion that can flee Ukraine in the future.

**Counterfactual analysis:** A baseline scenario, a status quo, represents a forecast of the conflict based on its current trajectory (as of 2022 April 21) and absent any exogenous shocks that would otherwise alter its dynamics. This forecast and its suggested impact on refugee outflows alone represent a valuable asset that could greatly assist humanitarians by informing resource staging and personnel management to prepare for anticipated refugees. Early reports from local officials in Poland providing humanitarian aid to Ukrainian refugees indicated that uncertainty about near-term future numbers of refugees represented a major concern, particularly in regard to the availability of housing accommodation as well as the ideal delivery location for limited food and medical resources (46). Alongside this baseline status quo scenario, we provide two plausible scenarios that would complement a policymaker’s contingency planning analysis. These scenarios represent outcomes that experts could plausibly have anticipated occurring at the end of April 2022.

*First counterfactual scenario—the Belarus Offensive.* This scenario represents a hypothetical second Russian offensive out of Belarus in late April penetrating as far as 100 km into Ukraine along the Belarusian border. After the initial Russian advance towards Kiev faltered in March a considerable Russian military force retreated back to staging grounds in Belarus. During the spring, concerns existed that these military assets could regroup and mount a second major offensive push on the Ukrainian capital (65) and these concerns persisted for the first year of the war featuring in intelligence assessments of potential future combat theaters (66). Therefore, this forecast considers the consequence of a second major Russian invasion from the north on refugee flows which would primarily originate from civilians fleeing violence near populated places surrounding the capital.

*Second counterfactual scenario—the Kherson Counteroffensive.* This scenario forecasts the conflict shifting from Ukraine’s east (where the conflict had concentrated by April 21—the cutoff date for data used to fit the conflict forecasting model) southward towards the Kherson Oblast. This scenario reflects conditions likely to emerge over a 2-week window if Ukraine had launched an earlier counteroffensive against Russian forces which had occupied Kherson and the surrounding settlements in the area along the Dnieper River. Kherson represented a key strategic port city in the south and, with its population under Russian occupation, represented a vital interest for Ukraine to recover. In late April speculation suggested that a Ukrainian effort to retake the city was potentially imminent (67) and policymakers, therefore, would have had an interest in understanding how such a major military action would have impacted the flow of refugees out of Ukraine (see Appendix SB for more detail on the conflict counterfactual and visualization of the spatial domains of these scenarios).

**Analysis and implications:** Table 1 summarizes the aggregate results of our forecast analysis on refugee outflows and also presents the total number of conflict events and fatalities forecast in the three scenarios as well as the observed numbers reported in ACLED data over an identical time period. The table provides estimates of the total estimated refugee outflows estimated in each of these three scenarios as well as the total number of estimated refugees computed using *observed* ACLED events reported over the forecast period (April 22–May 05). Based on ABSCIM aggregated refugee estimates, all three conflict forecast scenarios suggested higher overall levels of refugee outflows than the estimates generated using the observed data. Using *observed* conflict events reported by ACLED ABSCIM estimates 600 thousand refugees over this time period while with using the *forecast* conflict data the ABSCIM estimated an average 830.7 thousand refugees across the three scenarios. During the forecast period, border crossing checkpoints reported 650 thousand refugees leaving Ukraine. Therefore, based on this reported crossing figure, using observed conflict events the model underestimated refugee flows during the forecast period by ∼7% and using the forecast conflict events overestimated refugee flows by ∼28%.

**Table 1.** Estimates from scenario forecast analysis.

Scenario	Description	Events	Fatalities	ABSCIM
				estimates (1000 s)
Status quo	Forecast trend with no shock event	759	760	833.4
Belarus offensive	Russian offensive penetrating up to 100 km into Ukraine from Belarus border	1,034	719	828.5
Kherson counteroffensive	Ukrainian counteroffensive in Kherson and within 50 km of Oblast borders	1,073	987	830.3

∙ Refugees reported in border crossing data (April 22–May 05): 650,268

∙ Refugees estimated using *observed* ACLED events: 600,071

Three reasons help to account for this discrepancy. First, the conflict forecasts are probabilistic in nature and therefore sample conflict points across a wider geographic space thereby exposing more agents to violence under the reported scenario (see Appendix SB for more details on the conflict forecast). Second, the counterfactual scenarios simulate new conflict theatres opening in regions without past violence. This exposes a larger population that was previously more geographically distant from the conflict’s frontlines to conflict events leading to higher estimated outflows. Finally, the model’s treatment of internal displacement may lead to these higher figures within the context of more spatially dispersed events. Events occurring across a larger geographic domain likely lead to increased probability of agents migrating rather than fleeing internally (see Discussion section), suggesting a refinement opportunity in future research expanding on this approach by more explicitly incorporating internal displacement and sequential agent moves either again as internally displaced or as refugees.

**Fig. 3.**
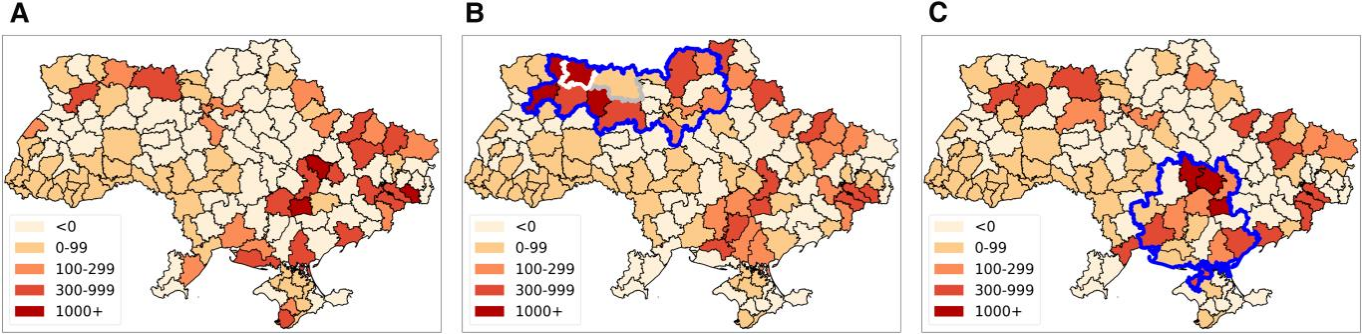
Visualization of spatial origin of fleeing Ukrainians in response to three possible conflict scenarios. To develop a better sense of the origin locations for refugees in these scenarios, we subtract the estimated Raion aggregates from the Raion aggregates estimated using the observed conflict data. (See Appendix SB for visualizing outflow difference with status quo.) The figures highlight the Raions most likely to produce greater refugee flows (darker red) under the given scenario relative to the observed estimates. In the status quo scenario in a), most Raions exhibit similar outflows as the outflow generated using observed conflict data. However, the few Raions with higher estimates primarily lie on the periphery of the conflict space and therefore these Raions likely did not have any observed conflict events but, owing to forecast uncertainty, did have events in the status quo scenario resulting in larger outflows. Under the Belarus Offensive scenario in b), the bulk of new refugees originate from Raions along or near the Ukrainian border with Belarus. In fact, Raions like Sarnenskyi and Zviahelskyi observe greater than 10 K refugee estimate differences under this scenario compared to the observed scenario. c) shows similar differences in outflow across Raions under the Kherson counteroffensive scenario where the Raions with large estimate differences are observed around the center of the offensive. a) Status quo. b) Belarus offensive, and c) Kherson counter-offensive.

Figure 3a summarizes the impact of the status quo forecast on the number of refugee outflows in terms of the increased difference per Raions relative to estimates produced using observed ACLED events. As anticipated, it can be observed that the hotspots appear primarily mostly along the southeastern part where most of the conflict events were observed. The majority of Raions in the status quo forecast exhibit no substantive differences relative to estimates using observed data. Overall, the status quo forecast performs well and for the majority of Raions in Ukraine captures similar migration dynamics relative to estimates produced using observed conflict events. This provides additional confidence in using the approach to assess contingencies such as the Belarus and Kherson scenarios.

Figure 3b presents refugee flows estimated to occur during a hypothetical Russian offensive originating from Belarus beginning on 2022 April 22, and penetrating up to 100 km into Ukraine over the 2-week forecasting period. Some interesting patterns emerge which highlight the utility of ABSCIM to identify the potential for new migration under counterfactual contingencies. Notably, Raions near the Kiev region have *lower* overall estimated refugee outflows than do nearby surrounding Raions. The conflict model suggests that the in this scenario most fighting would occur in proximity to Kiev. We identify household depletion as the cause of this disparity. For example, consider two Raions, Sarnenskyi and Korostenskyi (marked with white and gray borders in Fig. 3b, respectively) situated near the border but showing different behavior in terms of refugee outflows during the offensive scenario. The number of household agents who had not still moved (IDP or refugee) before April 22 substantively differed owing to past conflict in the region. The number of total household agents in Sarnenskyi was initially 113,742 and by April 21 only 703 household agents had undergone migration (99.38% household agents remained). Among the 140,627 household agents in Korostenskyi 105,112 had already fled by April 21, leaving only 25.11% household agents remaining. The depletion of household agents in Korostenskyi represents the main reason that we do not observe a significant difference in outflow from this Raion during the offensive scenario even though it lies close to the border. (For visualization of household depletion over time, see Appendix SB.) By providing such detailed information on the number of Ukrainians living in plausible future conflict spaces ABSCIM can effectively summarize the potential for future refugee waves under a new conflict scenario.

Finally, Fig. 3c produces equivalent estimates on the total number of refugees anticipated under the Kherson counteroffensive scenario. Interestingly, rather than Raions at the center of the counteroffensive closest to the city of Kherson, the Raions along the periphery of the Kherson Oblast (bold border) exhibit the largest total outflow relative to the outflows estimated by ABSCIM using the observed data. Again, these differences highlight the consequence of a more geographically diffused conflict scenario on total refugee flows. New conflict events occurring in periphery regions of the Kherson Oblast lead to higher overall outflows in a forecast that anticipates more expansive fighting in response to a Ukrainian initiative to retake the city of Kherson.

Overall, these analyses emphasize the fact that ABSCIM can generate refugee estimates for a variety of policy-relevant scenarios including a best (status quo) forecast of conflict events but also for contingencies that would assist policymakers in conducting a counterfactual analyses in response to unfolding conflict conditions and refugee flows. Furthermore, the granular nature of data estimated using ABSCIM which we have analyzed here at the Raion level allows us to disaggregate the results at any spatiotemporal resolution as well as along key demographic indicators such as age and gender. Such precision can greatly facilitate the staging of humanitarian assistance in anticipation of plausible contingencies or for particular refugee needs based on demographic traits or anticipated forms of violence a refugee may have experienced while fleeing the conflict.

## Discussion

Migration is a complex process and the potential drivers may be too expensive to be computationally quantifiable (40, 68). In this work, we take a step towards modeling conflict-induced migration from both a social and computational standpoint, in the context of the Russian invasion of Ukraine. Combining decision-making process of real-world digital twins motivated by social theory with real-world events with an ABM, we produce daily estimates of refugee outflow along key demographic dimensions, as well as identify spatial origins of anticipated refugee flows, either at Oblast or Raion levels. Generation of high-resolution information makes the model appropriate for addressing a variety of policy relevant questions. We have illustrated potential policy use of the ABM by analyzing wartime sexual assault and potential refugee flows under a variety of plausible future conflict scenarios. Furthermore, although limited “ground truth” data exist in the context of rapidly unfolding crisis situations such as the Ukrainian war during its initial weeks, the ABM was validated using best-reported estimates of daily border crossings, demonstrating the validity of the model; further bolstering the utility of the model as a tool for policymakers to employ in a crisis response workflow.

While the framework presented takes a number of important steps toward developing a computational model of conflict-induced displacement informed by social theory, several avenues exist to improve or otherwise expand on this initial work. First, once initially migrated, the ABM does not consider these agents in future migration. However, return migration and internally displaced civilians represent factors contributing to migration and should be incorporated into the framework. For example, future modeling could allow new conflict events to impact the likelihood that an internally displaced agent (one who has already fled internally in response to violence) subsequently decides to flee across an international border. Furthermore, tracking internally displaced using the ABM would provide additional valuable information as these individuals often represent a population most in need of humanitarian assistance yet most difficult to reach.

Second, our model employs a static peer effect over the course of the conflict whereas the trend of dynamic simulation can almost certainly give the peer effect a dynamic nature. In Layman’s terms, the degree to which each agent communicates and draws information from their neighbors can vary with the progress of time, which our model does not take into account. By allowing the peer effect to vary over time the model could more flexibly adjust to household depletion in areas heavily impacted by armed conflict. Substantively, this would translate to allowing agents to rationally update how they interpret the actions of their peers in these spaces. For example, rather than a fixed peer effect the model could incorporate an over-time adjustment which allows the composition of the agent’s peer network to expand as the conflict unfolds in order for the agent to assess the actions of more distant agents also in the conflict space in their flee/stay decision-making process.

Third, the model does not make any inference about the destination of the migrants, making it primarily applicable in estimating the initial displacement during the shock period, during which data scarcity is more prevalent and the nature of migration is less predictable. After this period, migration is driven by other aspects (e.g. return migration and cascading migration) which require knowledge about the destination of the migrants caused by the initial displacement. As mentioned in the literature review, there exist studies that have attempted to answer the question of destination given the number of displacements as input. Therefore, a natural extension of our work would be to create an end-to-end model that identifies both the displacement from the origin country and their destinations. Such a model has the potential to explore cascading effects and return migration after the initial displacement.

Finally, our model takes an agnostic perspective with respect to actors perpetuating the conflict events. Specifically, the model does not distinguish between Russian or Ukrainian-initiated conflict events. Previous conflict research has demonstrated the consequences of actor identity on the rational decision-making of civilians with respect to their clustering behavior in space and the ultimate destination, whether internally or internationally (Steele, 2019). Given Russian tactical choices to target civilian settlements within Ukraine, therefore distinguishing between Russian and Ukrainian-initiated acts could serve as a fruitful pathway to estimate conflict events, leading to internal displacement vs. conflict events more likely to contribute to outbound refugee flows.

Nonetheless, the model in its current form produces demographically detailed daily estimates of conflict-induced migration, validated using the best publicly available reporting of daily border crossings out of Ukraine during the initial weeks of the conflict. Furthermore, the model produces these estimates with minimal data inputs: the model requires only reported conflict events and a synthetic set of agents. Additionally, as we have demonstrated, geostatistical conflict forecasts can generate plausible events as inputs into the model for contingency planning analysis thereby underscoring the policy utility of the model presented here.

## Supplementary Material

pgae080_Supplementary_Data

## Data Availability

The source code, preprocessing scripts, and the border crossing data underlying this article are available in the Github Repository here. The conflict data can be accessed by opening an account through the ACLED portal and generating a key. The synthetic population data can be obtained from this link and can be used under CC by SA 4.0. The source for the IOM survey report used for validating demographic estimation is provided in the References of Appendix SB.
