# Supplementary material for: An agent-based framework to study forced migration: A case study of Ukraine
Source: PNAS Nexus. 2024 Mar 19;3(3):pgae080. doi: 10.1093/pnasnexus/pgae080 (PMC10949908; doi:10.1093/pnasnexus/pgae080)
Supplement: pgae080_Supplementary_Data [file pgae080_supplementary_data.zip › PNASNEXUS-PNASNEXUS-2023-00705R-s01.pdf]

Appendix SA for An Agent-Based Framework to Study Forced Migration: A Case Study of Ukraine

Model Design and other implementation Details

Zakaria Mehrab[1,2] Logan Stundal[1, 3] Srinivasan Venkatramanan[1]
Samarth Swarup[1] Bryan Lewis[1] Henning S. Mortveit[1]
Christopher L. Barrett[1, 2] Abhishek Pandey[4] Chad R. Wells[4]
Alison P. Galvani[4] Burton H. Singer[5] David Leblang[3] Rita R. Colwell[6]
Madhav V. Marathe[1, 2]

[1]Biocomplexity Institute & Initiative, University of Virginia
[2]Department of Computer Science, University of Virginia
[3]Department of Political Science, University of Virginia
[4]Center for Infectious Disease Modeling and Analysis, Yale School of Public Health
[5]Emerging Pathogens Institute, University of Florida
[6]Center for Bioinformatics and Computational Biology, University of Maryland

March 4, 2024

SA 1 Other Related Works

Ukraine war: Dijcke et al. [41] studied the responsiveness of Ukrainians to public alerts by studying geolocation data before and after alerts. They discover that the mobility pattern of civilians changes sharply after the alerts. However, this change gradually declines as the war progresses. They also perform counterfactual analysis to conclude that these alerts are highly effective in reducing casualties and would have been more effective if the responsiveness did not decline with time. Duszczyk et al. [15] assess the situation of refugees from Ukraine into Poland in the context of their integration into the labor market. They observe that although the current market has successfully accommodated the incoming migrants due to the high demand for workers, the situation may worsen due to the impending global economic downturn. Leasure et al. [24] used anonymized Facebook data to nowcast (estimate in real-time) the displacement amount from Ukraine at Oblast level. However, it is limited by both the availability and the spatial resolution. Moreover, this estimation is not modeled as a result of the drivers of migration (conflict events), making it infeasible for what-if scenario analysis. Stitcher et al. [37] reviewed types of satellite images to identify conflict events and concluded that these images are helpful in screening large conflict areas. Chumachenko et al. [13] studied the impact of Ukrainian migrants to Spain as a result of the invasion in the spread of COVID-19 using an ensembled based machine learning method and concluded that the refugee flow is not a decisive factor of the transmission of COVID-19 in Spain.

Apart from these, there are some agent-based modeling approaches mainly focused on different disease transmission in Ukraine in the past. For example, Chumachenko et al. [12] proposed an Agent-based model to model the spread of Syphilis in the Kharkiv region of Ukraine. Upon comparing with real data, they concluded that social factors contributed more to spreading of the

disease than medical factors. They had previously used ABM to simulate transmission of other diseases like Hepatitis B [10] or HIV [11].

Finally, a large body of works are more focused on suggesting policies regarding the incoming refugees from Ukraine. Bahar et al. [3] proposed that the policies directed towards Ukrainian refugees in the host countries should be welcoming rather than disbarring, as the incoming refugees can become a useful addition to the economy. The suggestion of Quinn et al. [31] complements the previous work by indicating that a long-term migration is likely to occur and policymakers should accomodate these refugees and permanent resettlement is likely to follow.

Traditional model for Migration (non-ABM) A number of models have been proposed to model normal migration between regions. The gravity model [42] assumes that movement between two locations is proportional to the populations in those regions and inversely proportional to their geographical distance. The radiation model [36] leverages the *intervening opportunities* to more accurately represent the flow of population from a source region to a destination. There have also been attempts to model migration using a machine learning-based approach [32]. However, the traditional models have specific forms, making them constrained in using data from different sources. On the other hand, a machine learning-based approach may bypass such constraints as new data can simply be used as new features but suffers from other problems like interpretability and fairness.

ABM in Migration: Lin et al. [25] proposed an agent-based approach to understand migration by using source destination features and using countries as agent through OLS regression. Although their approach is agent-based, it is more mesoscopic than microscopic. Klabunde et al. [22] try to identify key criteria behind the decision-making theory an agent-based model should have. Searle et al. [33] provide a conceptual framework from an abstract level. Both these works lack the necessary generalized mathematical formulation and a more concrete concept about the necessary input/outputs of such a model. Hattle et al. [19] studied the distribution of Syrian refugees into European countries and Turkey using an ABM. Frydenlund et al. [16] provided a prototype of agent-based model to predict IDP in Congo and suggested future directions for extending such a model. However, their agents represent only a fraction of the population, thereby likely to suffer from sampling issue. In the main document, we had cited a work by Suleimenova et al. [38] who had developed the FLEE model, which, given the conflict events and the intending refugees, is able to infer the distribution of these refugees to various destinations. Flee has also been used in assessing refugee distribution from Norhtern Ethopia to Sudan [39]. However, the availability of the intending refugees as a input poses a limitation (which our model estimates). They later approach to address this [9], with limited success. Another work [17] attempts to extend to make this model scalable. Hassani-Mahmooei et al. [18] studied the future of migration situation of migration in Bangladesh under climate change by utlizing ABM and observed that a large number of migrants are likely to move to eastern or northern districts which are less likely to be affected by drought or flood. [20] developed an ABM to study migration route resulting from information exchange between agents. They find that high exchange of information often results in agents finding routes resulting from social coordination, which is often suboptimal compared to the solution that can be achieved through self-exploration. Silveira et al. [35] developed a computational agent-based model to study rural-urban migration during industrialization and observed the equillibrium of expected wages in both sectors.

Social Theories for Migration: Firstly, there have been a set of works that advocates push and pull factors in the context of migration. Push factors like conflict events or loss of wealth [2] or environmental hazard [34] were identified as reasons that may drive individuals from their home. On the other hand, pull factors like economic prosperity or linguistic similarities [25, 4] of a place may attract these migrants such places. Among other theories, Dekker et al. [14] studied how herd

Table SA1: Summary of input attributes and sources

Entity	Attribute	Source
Conflict	T_j	Event time from ACLED
	L_j	Event lat/long from ACLED
	F_j	Fatalities from ACLED
Agent	$x_i(t)$	Household location of the agent from synthetic household data

behavior drives migration by exploring the effect of Social media through interviewing 54 Syrian refugees. In essence, herd behavior can be perceived similar to the *Subjective Norm* construct of the *Theory of Planned Behavior*. However, similar to other studies that present social theories of migration, a computation framework founded by such theories is absent.

We have also attempted several other contributions to the literature through some relevant parallel efforts. In one of these works, we focused on the interdisciplinary collaboration of Computer Science and Social Science to solve such a problem, assessed the generalizability of such a model, and highlighted the policy implication by conducting a case study of entrapped population [27]. In another work, we proposed a framework based on network agency that takes a network-first approach to understanding the behavior of a social system undergoing external events [28]. We conceptualize society as a multi-layered hierarchical entity and assume relationships within and across hierarchies. We extend this framework, to model the process of migration through a perception-action loop whilst taking network effects like peer influence into account. Sensitivity analysis of the model reveals the importance of peer influence in decision-making, emphasizing the significance of taking a network-first approach in the model formulation.

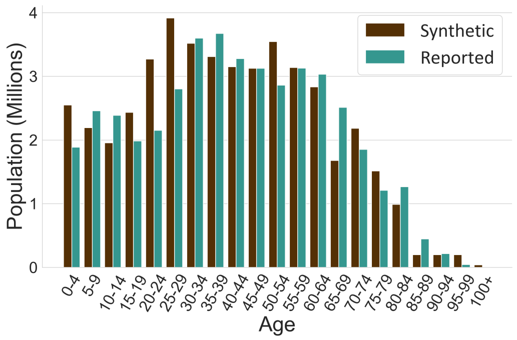

(a) Population pyramid

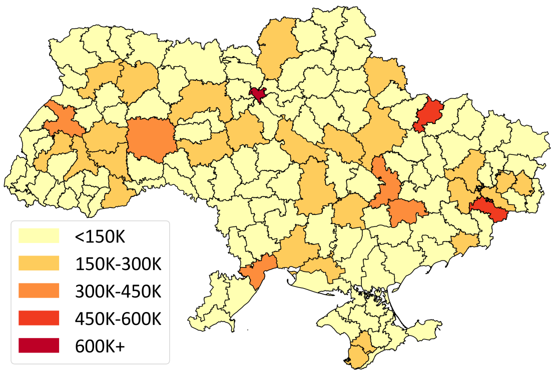

(b) Synthetic households

Figure SA1: Statistics about synthetic population for Ukraine. Figure SA1a shows the population age pyramid for Ukraine. The population age pyramid shows the age structure by distributing the population along the y-axis and the population is broken down into 5-year age groups represented as horizontal bars along the x-axis, with the youngest age groups at the left and the oldest at the right. For context, the population distribution by age groups of the synthetic population is displayed with population statistics of 2020 gathered from PopulationPyramid. Figure SA1b shows the distributions of households across Raions of Ukraine, with darker shaded Raions representing Raions with a larger number of households.

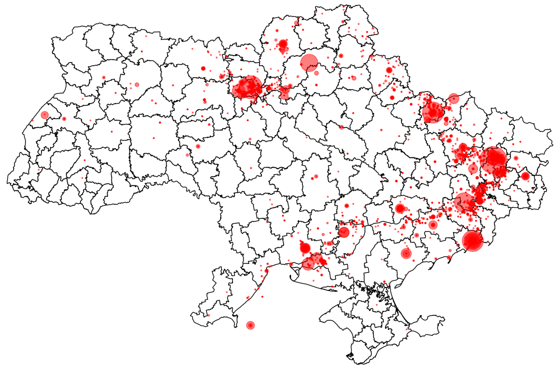

(a) Conflict location

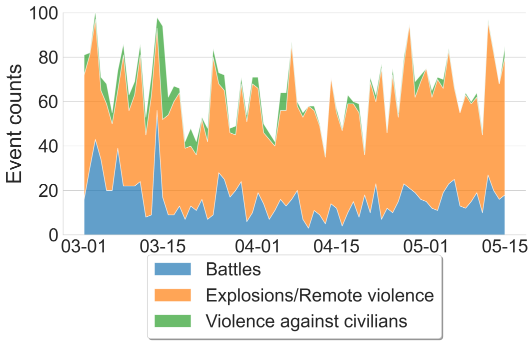

(b) Conflict trend

Figure SA2: Statistics about conflict events in Ukraine from March 1, 2022 to May 15, 2022 collected from ACLED. This time period encompasses our period of study and can be considered the shock period of the war. Figure SA2a shows the spatial distribution of the events during this time period. The events are heavier towards the eastern side where the Russian force began their invasion during the early period of the war. Figure SA2b shows daily trends of these types of conflict events likely to affect migration intent significantly.

Table SA2: Weights of different types of events

**Event Type**	W_j
Explosions	3
Battles/Violence	2
Protest/Riots	1
Others	0

SA 2 Data Utilization

This section describes the data used in the ABM, their sources and the use of these attributes from these sources to the model. A summary of the data sources is given in Table SA5.

SA 2.1 Conflict Data

In our model, we need information about the impact, time, and location of each conflict event. Obtaining the time and location information from ACLED is straightforward. The impact of an event is calculated as $I_j = w \times W_j \times F_j$, where w is a scaling parameter associated with the weight of conflict events, F_j is the fatality associated with event c_j, and W_j is a weight associated with the event whose value depends upon the type of the event. The weights are chosen based on the strengths defined in the GDELT-CAMEO codebook based on the displacement-generation effect for each event [6] and are shown in Table SA2.

SA 2.2 Agent Data

. We process the synthetic individual data to convert each individual to one of the four following demographic groups:

1. Elderly: If their age is above 60
2. Child: If their age is below 18

Table SA3: Flee Bias of agents according to their demography

Group	B_i
Elderly	0.15 (without family)<br>0.2 (with family)
Child	0.5 (without family)<br>0.7 (with family)
Adult Male	0.02 (without family)<br>0.05 (with family)
Adult Female	0.2 (without family)<br>0.7 (with family)

3. Adult Male: If their age is between 18 to 60 and their gender is male.

4. Adult Female: if their age is between 18 to 60 and their gender is female.

We also look at the household of each individual to classify each individual as part of a family or not. We assume that if a household contains more than one individual, the individual is part of a family. An agent's *risk-proneness* is defined as $\beta_i = b \times B_i$ where B_i is termed as the flee bias of the agents and b is a parameter for scaling these weights. The values are shown in Table SA3 and were chosen from studies including [7, 23, 8, 5]. For simplicity, we also assume that agents are always present in their households unless they decide to migrate. Therefore, we assume that the location of a person agent a_i is the same as the location of household ηa_i. The variables directly obtained from these data sources is presented in Table SA1.

SA 2.3 Border Crossing Data

To adopt our model to estimate the number of border-crossing, we add an additional layer of computation to our model. Specifically, we assume that each agent who wants to migrate may cross the border with a probability of λ or may be internally displaced with a probability of $1 - \lambda$. Specifically, if a household decides to migrate, then the individuals of that household may cross the border with a probability of λ. We acknowledge that similar to how social theory plays out behind the decision of migration as a result of conflict events, there are most certainly other theories involved to decide whether a potential migrant will be internally displaced (IDP) or cross borders to take refuge in a different country.

As a primary step, we simplify this to a ratio-based approach. Leveraging historical data on conflict-induced displacement from UNHCR [40] we estimate the mean (0.334) and standard deviation (0.04) of the ratio of refugees to IDPS since 2010. Using this mean and standard deviation of historical refugee/IDP ratios, during each step of the ABM we sample from a normal distribution the probability that a household will migrate at timestep t. While this approach relies upon historical migration responses to distinguish between refugees and IDPS, future researchers take alternative approaches such as by identifying the factors behind whether an individual decides to flee as an IDP or refugee. Furthermore, future work could expand here to include a third category of civilian not captured with this approach: individuals trapped in conflict spaces. These individuals represent the population in conflict intense regions that may get entrapped during migration and likely stay in vicinity of their households (e.g. basement of their household). But, these people intend to leave their home as a result of conflicts and, therefore, are part of the population that is produced as the final output. A future extension of the model is to take this entrapped population into account.

Table SA4: Summary of notations and symbols

Type	Symbol	Name	Additional Description
Set	C	Set of conflict events	An input to the model
Entity	c_j	j^{th} event	$c_j \in C$
Set	A	Set of person agents	An input to the model
Entity	a_i	i^{th} person agent	$a_i \in A$
Set	H	Set of household agents	An input to the model
Entity	h_k	k^{th} household agent	$h_k \in H$
Function	η	Mapping from person agent to household agent	An input to the model
Inverse image	η^{-1}	inverse image of η	Given a household agent, returns the set of person agents living in that household
Attribute	F_j	Fatality of event c_j	Attribute associated with a conflict event representing fatalities caused by that event
Attribute	T_j	Time of event c_j	Attribute associated with a conflict event representing the time of that event
Attribute	L_j	Location of event c_j	Attribute associated with a conflict event representing the location of that event. Can be represented by lat/long or other geometric representations.
Attribute	$x_i(t)$	Location of a_i at time t	Location associated with an agent at timestep t. Represented by lat/long representation.
Parameter	τ	Temporal decay parameter	Controls the temporal aspect for the observed impact of an event on an agent. A higher value of this parameter makes events that happened in the past less effective. $\tau > 1$.
Parameter	δ	Spatial decay parameter	Controls the spatial aspect for the observed impact of an event on an agent. A higher value of this parameter makes events at larger distances less effective. $\delta > 1$.
Parameter	θ	Memory decay parameter	Controls how much of the past event is retained by a not-yet migrated agent. $0 \leq \theta \leq 1$.
Parameter	Q	No-risk migration	Controls the probability that an agent migrates if they are not impacted by an event.
Parameter	v	Growth rate	Controls how increase in observed impact affects the migration probability
Parameter	b	Bias scale	Scaling parameter to control agent's risk-proneness
Parameter	w	Conflict scale	Scaling parameter to control intensity of an event
Parameter	I_{lo}	Low-neighbor threshold	An agent who wants to migrate will not migrate if less than I_{lo} of their neighbors are migrating.
Parameter	I_{hi}	High-neighbor threshold	An agent who did not decide to migrate will migrate if more than I_{hi} of their neighbors are migrating.
Hyperparameter	W_j	Weight of event c_j	Weight based on the type of event. Refer to Table SA2.
Hyperparameter	B_i	Flee bias of agent a_i	Flee bias based on the demographic attribute of an agent. Refer to Table SA3.

Table SA5: Summary of Data Sources

Data	Source	Purpose
Conflict data	ACLED [1]	Input to the model
Agent data	BII Synthetic Population data [29]	Input to the model
Border-crossing data	HDX [21]	Calibration and Validation

SA 3 Parameter calibration

Our model has a few parameters. Some of them were chosen as hyper-parameters. The other parameters need to be calibrated so that they can generate sensible estimations of the number of migrants. Various calibration techniques can be used to calibrate agent-based models. We employ the co-ordinate descent method as our technique.

Coordinate descent is an iterative algorithm that minimizes a function by minimizing it one direction at a time through several rounds. Let $F(X)$ be the objective function where $x \in R^n$. Starting with the initial values $X^0 = (x_1^0, x_2^0, ..x_n^0)$, the algorithm takes each variable at a time and tries to minimize the objective function for that variable only while keeping the other variables fixed. Therefore, each round consists of n steps where in one step the algorithm solves a single variable optimization problem. Formally, at i^{th} step of round k, we try to solve the following single variable optimization problem for finding x_i^k as

$$x_i^k = \underset{y \in [L_i, R_i]}{\arg\min} F(x_1^k, ..x_{i-1}^k, y, x_{i+1}^{k-1}, ..x_n^{k-1}) \ . \tag{1}$$

For solving the single variable optimization problem, we employ a grid search method over a pre-specified $[L_i, R_i]$ range for each variable.

In our case, let $F(\Theta_{ABM}) = \{\hat{y}(t_1), \hat{y}(t_2), ...\}$ be the vector of border-crossing estimated by our model and $Y = \{y(t_1), y(t_2), ...\}$ be the vector of observed border-crossing obtained from HDX. Here, $\hat{y}(t_i)$ and $y(t_i)$ represent the estimated border-crossing by the model and the observed border-crossing at timestep t_i, respectively. Simultaneously, $\Theta_{ABM} = (\Theta_1, \Theta_2, ...)$ encapsulates all parameters of the agent-based model. With this, we calibrate our model at i^{th} step of round k as follows.

$$\Theta_i^k = \underset{z \in [L_i, R_i]}{\arg\min} ||F(\Theta_1^k, ..\Theta_{i-1}^k, z, \Theta_{i+1}^{k-1}, ...) - Y||_2^2 \tag{2}$$

It is to be noted that, both $F(\Theta_{ABM})$ and Y are of the same sizes and the timesteps for the estimated border-crossing vector, $F(\Theta_{ABM})$ are consistent with the timesteps in Y.

We perform the calibration for two rounds. A list of all the parameters and their range for performing the grid search is provided in Table SA6. Apart from the parameters specified in this table, there are a few other non-calibrated parameters that were put as additional constraints which we will describe in the next section.

SA 4 Implementation Details

Our simulation runs for each day between March 1, 2022 and May 15, 2022. Since this is the shock period of the war in Ukraine, we think this period is of utmost importance to study. The conflict data consists of 22,021 events throughout this period and the agent data consists of 19M households agents and around 45M person agents. The entire simulation takes around 3-4 hours to complete.

Table SA6: Parameter Calibration

Index	Notation	Name	Initial range	Final value
Θ_1	τ	Temporal decay	$(1.0, 2.0]$	1.02
Θ_2	δ	Spatial Decay	$(1.0, 10.0]$	2.212
Θ_3	θ	Memory decay	0.9867 [30]	0.9867
Θ_4	Q	No-risk migration	$[1.0, 100.0]$	55.0
Θ_5	v	Growth rate	(0.0,10.0]	0.253
Θ_6	b	Bias scale	(0.0,1.0]	0.25
Θ_7	w	Conflict scale	(0.0,1.0]	0.34
Θ_8	I_{lo}	Low-neighbor threshold	[0,5]	0
Θ_9	I_{hi}	High-neighbor threshold	[5,30]	5

While agents consider all the past events, the outflow at time t is mapped to the conflict events of $t-7$ to $t-18$ days to account for travel times. This translates to the following assumption that an agent considers events in the past 18 days and takes 7 days to travel out of Ukraine if they decide to migrate, a reasonable assumption agreed upon by the authors. We acknowledge that the travel time of a refugee is dependent on a multitude of factors, including the distance from their home location to the border point they want to cross, the path they take and the travel modes. However, these factors are mainly associated with the destination chosen by a refugee and determining that is out of the scope of the current study. Therefore, we choose a single constant for the travel time. However, we plan on addressing this further in future extensions of the model.

In order to consider peer effect, we construct a household network $G_H(V_H, E_H)$ where E_H follows the edge construction scheme that a pair of households $(h_\alpha, h_\beta) \in E_H$ if and only if h_α and h_β both are under the same S2 level 13 cell. We choose this scheme because previously it has been used for representing neighboring points of interest [26] and level 13 cells have an area of roughly one square kilometer; a reasonable size for considering a neighborhood. The two values for the peer effect threshold functions are $I_{lo} = 1$ and $I_{hi} = 5$. For efficient computation, we run the model parallelly across different computing nodes where each computing node simulates migration intent for agents in one particular Raion. We also limit the agents of each Raion to interact with the conflict events in that Raion only. However, we also want to relax the interaction to be bounded by artificial borders created for tax purposes. Therefore, we create a 5 km buffer around each Raion border so that agents can interact with the conflict events around the neighboring Raion to some extent. Moreover, 97% of the conflict events used in our analysis are recorded with precision at the town or region level. Therefore, this small buffer is sufficient to reduce any noise associated with the location of the events. The observed border crossing data contains border crossing for 60 data points (days). Among these, we randomly select 15 data points (25% of the observed points) and calibrate our ABM model for those 15 data points.

SA 5 Miscellaneous statistics about Migrant Outflow

Figure SA3a and SA3b show the ABM estimates alongside the border crossing data, with no rolling average and 2-day rolling average applied. The large difference in the two plots indicates that the border crossing data is most likely very noisy. However, it is still interesting how the ABM is trying to capture some of the early spikes. Figure SA3c shows the refugee outflow broken down by various age groups. Figure SA3d and Figure SA3e disaggregate it further by female and male gender, respectively. Similar in trend, male outflow is a little bit less than half compared to the female

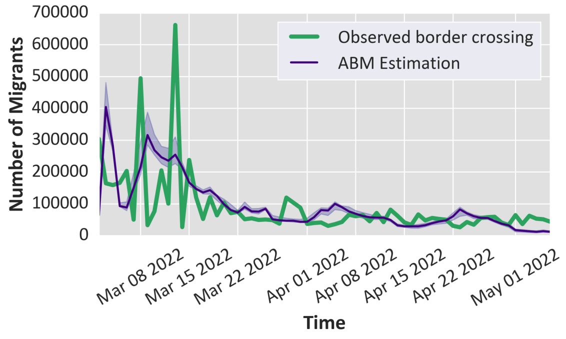

(a) Daily migrants (no smoothing applied)

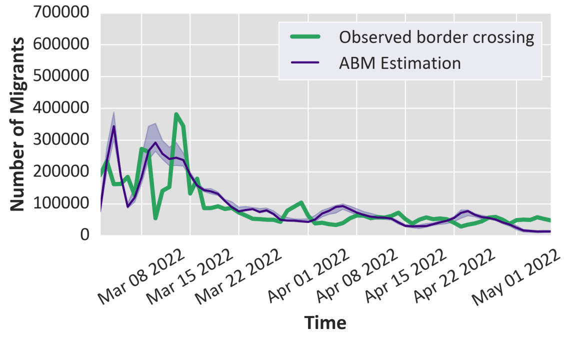

(b) Daily migrants (2-day average)

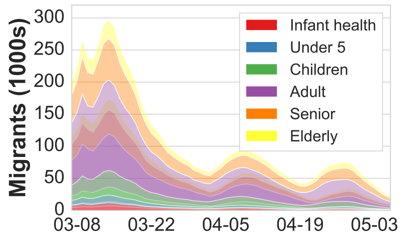

(c) Age (all)

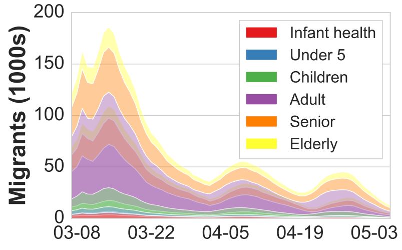

(d) Age Breakdown for Female Migrants

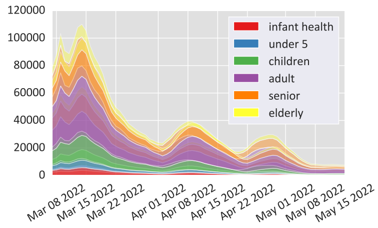

(e) Age Breakdown for Male Migrants

Figure SA3

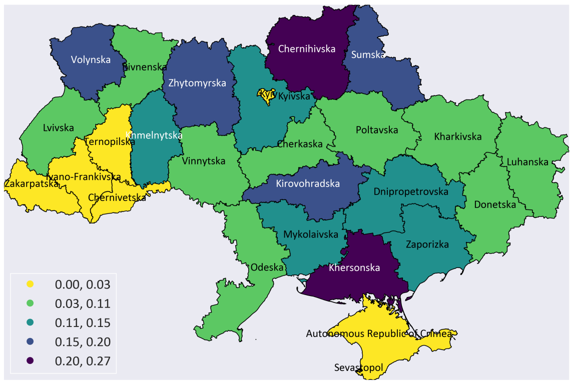

(a) Oblast level

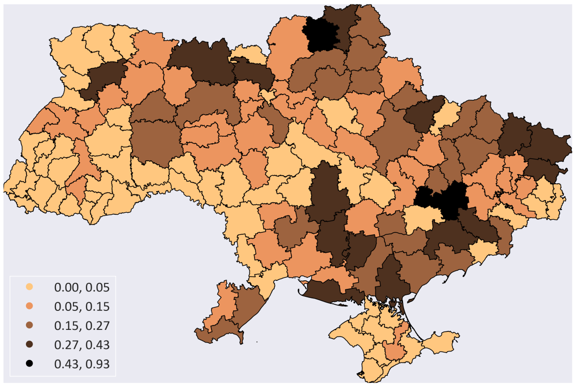

(b) Raion level.

Figure SA4: Spatial-subnational ratio estimates of refugee with respect to total population of the ABM.

migrants in terms of magnitude. Figure SA4 shows the ratio of refugee estimates with respect to total population at Oblast and Raion level.

Table SA7: Metric Comparison - Ukraine

Metric	ABM (Mean ± Stddev.)	LinReg
PCC	0.947 ±0.004	0.81
RMSE	20078.716 ±2343.72	34957.185
MAPE	0.255 ±0.016	0.3962

Table SA8: Metric Comparison - Northern Mali

Metric	ABM (Mean ± Stddev.)	LinReg
PCC	0.974 ± 0.003	0.68
RMSE	18601.007 ±3539.115	28763.8274
MAPE	0.173 ±0.085	0.3411

SA 6 Comparison with Vanilla Regression

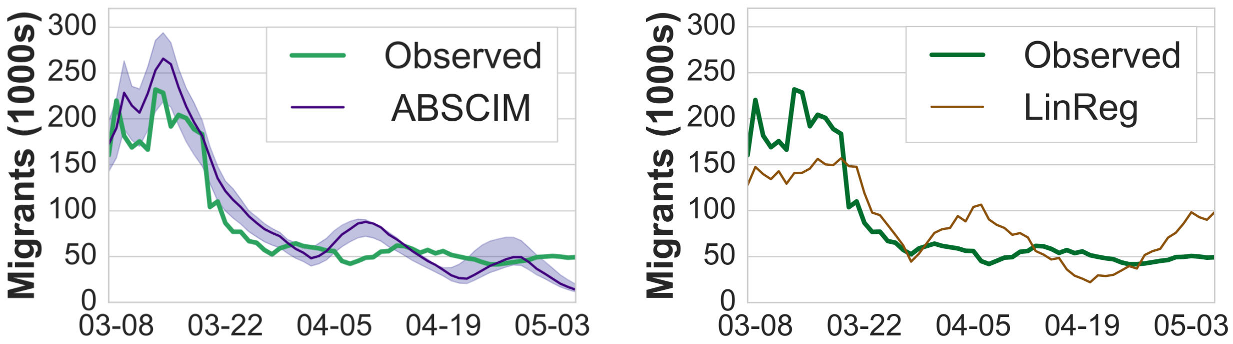

(a) ABM Estimation

(b) Vanilla Regression Estimation

Figure SA5: Ukraine

In order to develop a sense of how good of a fit our ABM is with respect to the observed scenario, we fit a vanilla regression as a baseline method, following [38]. The regression was trained with conflict information of the past seven days in order to predict the refugee outflow at the current day. This is consistent with the number of conflict days processed by our ABM. Since using each individual conflict event as an individual feature will blow up the number of features used by a regression method, we use the total number of events and the total number of fatalities as features, giving us a total of 14 features, two for each day. The regression model was trained for all the available datasets and then Figure SA5b shows the in-sample prediction of the regression method. From the Figure and also from Table SA7, it is evident that ABM performs better than the vanilla regression method both in terms of capturing the trend (PCC) and the scale (RMSE and MAPE) of the daily estimation of refugees.

SA 7 Generalizability and Humanitarian Crisis Feasibility

In line with discussion on how our model can be used in different conflict settings, with some of the parameters requiring re-calibration, we fit the ABM to estimate the migration outflow during the 2012 Northern Mali scenario. This model used the conflict data for Mali from March 1, 2012 to

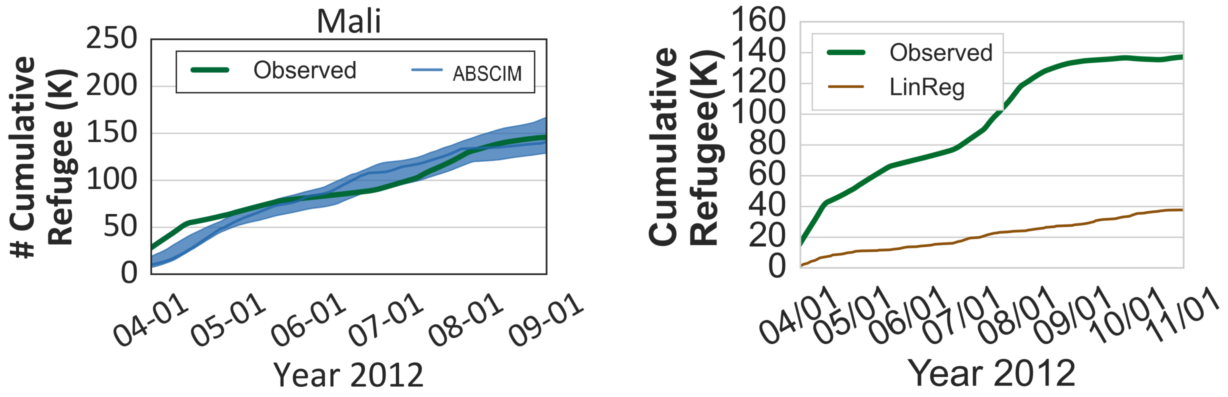

(a) ABM Estimation

(b) Vanilla Regression Estimation

Figure SA6: Northern Mali

October 31, 2012 to perform the daily estimation. Mali in 2012 represents an ideal additional case within which to test the model as the context of the Mali conflict deviates from the Russian invasion of Ukraine. Beginning in 2012 year internal conflict in Mali greatly expanded as non-governmental militant groups fighting for the independence of the Azawad region of Northeast Mali significantly escalated hostilities with the Malian government and its military forces. Over the course of 2012 this internal armed conflict expanded to include several non-state military groups pitted against the state encompassing a wide geographic area thereby exposing civilians to violence. By selecting an internal armed conflict here we are able to demonstrate the model's utility for capturing conflict-induced migration in various conflict contexts.

To calibrate this model, we kept the distance decay parameters and the scaling parameters unchanged from their nominal value for the Ukraine scenario. The other parameters were recalibrated using the method outlined in SA 3. To calibrate, the model was simulated in an HPC cluster limited by the total number of nodes (40) and memory (384 GB) available simultaneously. By running the model for each admin2 region parallelly on the cluster machines, the recalibration took around 2 days.

The re-calibrated model was used to estimate the daily refugee outflow and the result is shown as daily cumulative refugee in Figure SA6a, smoothed by a two-week rolling average. We also compare with a linear regressor trained on past two-week conflict information to compare the estimation. From Table SA8, we can see that our model again outperforms the regressor in all avenues. This underscores the generalizability potential of our model and the potential to use this in a different conflict setting.

The recalibrated parameters were as follows: $Q = 1.3$ (55 for Ukraine), $v = 0.983$ (0.253 for Ukraine), $I_{lo} = 1$ (0 for Ukraine) and $I_hi = 30$ (5 for Ukraine). The recalibrated value of Q suggests that people are more likely to migrate in this setting even if they did not experience any violence personally. A higher value of v also indicates that the intention to migrate increases steeper with an increase in violence, compared to the rate we obtained for Ukraine. However, the high value of I_{hi} is surprising and there can be two reasons for this. First, the people do not migrate unless many of their neighbors migrate, in which case most of the migration will be very spatially concentrated. This is a likely case since most of the conflicts were concentrated on the Northern side. Second, the high value of I_{hi} makes many less concentrated neighborhoods less likely to ever migrate. To fit the observed data, the model may have focused on concentrated zones where conflicts were

prevalent and kept other zones robust to migration. Further investigation is required to validate these hypotheses.

References

[1] ACLED. Introducing ACLED-Armed Conflict Location and Event Data. *Journal of Peace Research*, 47(5), 2010. 7

[2] Prakash Adhikari. Conflict-induced displacement, understanding the causes of flight. *American Journal of Political Science*, 57(1):82–89, 2013. 2

[3] Dany Bahar, Chris Parsons, and Pierre-Louis Vezina. Countries should seize the opportunity to take in ukrainian refugeesit could transform their economies. 2022. 2

[4] Fabian Barthel and Eric Neumayer. Spatial dependence in asylum migration. *Journal of Ethnic and Migration Studies*, 2015. 2

[5] Zuzanna Brunarska and Artjoms Ivlevs. Family Influences on Migration Intentions: The Role of Past Experience of Involuntary Immobility. *Sociology*, 2022. 5

[6] Marcello Carammia et al. Forecasting asylum-related migration flows with machine learning and data at scale. *Scientific Reports*, 12(1), 2022. 4

[7] Jørgen Carling and Kerilyn Schewel. Revisiting aspiration and ability in international migration. *Journal of Ethnic and Migration Studies*, 44(6), 2018. 5

[8] Francesco Castelli. Drivers of migration: why do people move? *Journal of travel medicine*, 25(1), 2018. 5

[9] N Chan, Diana Suleimenova, David Bell, and Derek Groen. Modelling refugees escaping violent events: a feasibility study from an input data perspective. In *Proceedings of the Operational Research Society 9th Simulation Workshop, Worcestershire, England*, 2018. 2

[10] Dmytro Chumachenko and Tetyana Chumachenko. Agent-based model of the epidemic process of diseases with multiple routes of infection transmission development and evaluation. 2016. 2

[11] Dmytro Chumachenko and Tetyana Chumachenko. Agent-based simulation of HIV epidemic social impact in Kharkiv region of Ukraine. 2017. 2

[12] Dmytro Chumachenko et al. Development of an intelligent agent-based model of the epidemic process of syphilis. In *Proc. of CSIT*, 2019. 1

[13] Dmytro Chumachenko, Tetiana Pudkina, and Tetyana Chumachenko. Assessing the impact of the russian war in Ukraine on COVID-19 transmission in Spain: a machine learning-based study. 2023. 1

[14] Rianne Dekker, Godfried Engbersen, Jeanine Klaver, and Hanna Vonk. Smart refugees: How Syrian asylum migrants use social media information in migration decision-making. *Social Media+ Society*, 4(1):2056305118764439, 2018. 2

[15] Maciej Duszczyk, Agata Górny, Paweł Kaczmarczyk, and Andrzej Kubisiak. War refugees from Ukraine in Poland–one year after the Russian aggression. Socioeconomic consequences and challenges. *Regional Science Policy & Practice*, 15(1):181–199, 2023. 1

[16] Erika Frydenlund et al. Where are they headed next? Modeling emergent displaced camps in the DRC using agent-based models. In *Proc. of WSC*. IEEE, 2018. 2

[17] Derek Groen. Development of a multiscale simulation approach for forced migration. In *Computational Science–ICCS 2018: 18th International Conference, Wuxi, China, June 11-13, 2018, Proceedings, Part II 18*, pages 869–875. Springer, 2018. 2

[18] Behrooz Hassani-Mahmooei and Brett W. Parris. Climate change and internal migration patterns in Bangladesh: an agent-based model. *Environment and Development Economics*, 17, 2012. 2

[19] Anna Hattle, Katherine Shulin Yang, and Sicheng Zeng. Modeling the Syrian Refugee Crisis with Agents and Systems. *UMAP Journal*, 37(2), 2016. 2

[20] Martin Hinsch and Jakub Bijak. Rumours lead to self-organized migration routes. 2019. 2

[21] HUMDATA. The Humanitarian Data Exchange . https://data.humdata.org/. [Online; accessed December 2, 2022]. 7

[22] Anna Klabunde and Frans Willekens. Decision-making in agent-based models of migration: state of the art and challenges. *European Journal of Population*, 32(1), 2016. 2

[23] G Lanau. Migration drivers: Why do people migrate. *EU-Logos Athéna*, 2019. 5

[24] Douglas R Leasure, Ridhi Kashyap, Francesco Rampazzo, Claire A Dooley, Benjamin Elbers, Maksym Bondarenko, Mark Verhagen, Arun Frey, Jiani Yan, Evelina T Akimova, et al. Nowcasting daily population displacement in Ukraine through social media advertising data. *Population and Development Review*, 2023. 1

[25] Larry Lin, Kathleen M Carley, and Shih-Fen Cheng. An agent-based approach to human migration movement. In *Proc. of WSC*. IEEE, 2016. 2

[26] Zakaria Mehrab et al. Data-driven real-time strategic placement of mobile vaccine distribution sites. 36(11):12573–12579, 2022. 8

[27] Zakaria Mehrab, Stundal Logan, Srinivasan Venkatramanan, Samarth Swarup, Henning Mortveit, Christopher Barrett, Abhishek Pandey, et al. A generalizable theory-driven agent-based framework to study conflict-induced forced migration. In *Proceedings of the 34th AAAI Conference on Artificial Intelligence - Accepted (To appear)*, 2024. 3

[28] Mehrab, Zakaria, Stundal Logan, Srinivasan Venkatramanan, Samarth Swarup, Henning Mortveit, Christopher Barrett, Abhishek Pandey, et al. Network agency: An agent-based model of forced migration from ukraine. In *Proceedings of the 23rd International Conference on Autonomous Agents and Multiagent Systems. - Accepted (To appear)*, 2024. 3

[29] Henning. S Mortveit et al. Synthetic Populations and Interaction Networks for the U.S. Technical report, University of Virginia, 2020. NSSAC Technical Report: #2019-025. 7

[30] Rafael Prieto Curiel and Steven Richard Bishop. Fear of crime: the impact of different distributions of victimisation. *Palgrave Communications*, 4(1), 2018. 8

[31] Simon Quinn and Isabel Ruiz. Forced migration: evidence and policy challenges. *Oxford Review of Economic Policy*, 38(3):403–413, 2022. 2

[32] Caleb Robinson and Bistra Dilkina. A machine learning approach to modeling human migration. In *Proc. of SIGCAS*, 2018. 2

[33] C Searle and Jan H van Vuuren. Modelling forced migration: A framework for conflict-induced forced migration modelling according to an agent-based approach. *Computers, Environment and Urban Systems*, 85, 2021. 2

[34] Brandie Banner Shackelford et al. Environmental health in forced displacement: a systematic scoping review of the emergency phase. *Science of the Total Environment*, 714:136553, 2020. 2

[35] Jaylson J. Silveira, Aquino L. Espíndola, and T. J. P. Penna. Agent-based model to rural–urban migration analysis. *Physica A: Statistical Mechanics and its Applications*, pages 445–456, 2006. 2

[36] Filippo Simini et al. A universal model for mobility and migration patterns. *Nature*, 484(7392), 2012. 2

[37] Valerie Sticher, Jan D Wegner, and Birke Pfeifle. Toward the Remote Monitoring of Armed Conflicts. *PNAS Nexus*, page pgad181, 2023. 1

[38] Diana Suleimenova, David Bell, and Derek Groen. A generalized simulation development approach for predicting refugee destinations. *Scientific reports*, 7(1), 2017. 2, 10

[39] Diana Suleimenova, William Low, and Derek Groen. An agent-based forced displacement simulation: A case study of the tigray crisis. In *Computational Science – ICCS 2022*, 2022. 2

[40] UNHCR. Population Figures. Technical report, United Nations, Jun 2022. 5

[41] David Van Dijcke, Austin L Wright, and Mark Polyak. Public response to government alerts saves lives during Russian invasion of Ukraine. *Proceedings of the National Academy of Sciences*, 120(18):e2220160120, 2023. 1

[42] George Kingsley Zipf. The P 1 P 2/D hypothesis: on the intercity movement of persons. *American sociological review*, 11(6), 1946. 2
