# Supplementary material for: An agent-based framework to study forced migration: A case study of Ukraine
Source: PNAS Nexus. 2024 Mar 19;3(3):pgae080. doi: 10.1093/pnasnexus/pgae080 (PMC10949908; doi:10.1093/pnasnexus/pgae080)
Supplement: pgae080_Supplementary_Data [file pgae080_supplementary_data.zip › PNASNEXUS-PNASNEXUS-2023-00705R-s02.pdf]

Appendix SB for An Agent-Based Framework to Study Forced Migration: A Case Study of Ukraine

Supplementary analysis

Zakaria Mehrab[1,2] Logan Stundal[1, 3] Srinivasan Venkatramanan[1]
Samarth Swarup[1] Bryan Lewis[1] Henning S. Mortveit[1]
Christopher L. Barrett[1, 2] Abhishek Pandey[4] Chad R. Wells[4]
Alison P. Galvani[4] Burton H. Singer[5] David Leblang[3] Rita R. Colwell[6]
Madhav V. Marathe[1, 2]

[1]Biocomplexity Institute & Initiative, University of Virginia
[2]Department of Computer Science, University of Virginia
[3]Department of Political Science, University of Virginia
[4]Center for Infectious Disease Modeling and Analysis, Yale School of Public Health
[5]Emerging Pathogens Institute, University of Florida
[6]Center for Bioinformatics and Computational Biology, University of Maryland

February 13, 2024

SB 1 Sexual violence extended analysis

In order to estimate the prevalence of sexual violence to have occurred during the initial period of conflict within Ukraine we employ estimates of the prevalence of wartime sexual assault compiled by Vu et al. (2014) [5]. We pair these sexual assault estimates with the demographically detailed output of ABSCIM to compute best estimates of Ukraine wartime sexual assaults given data limitations and reporting obstacles that prevent more accurate data collection by humanitarian organizations within the active conflict space.

The purpose of this supplemental analysis is twofold. First, by incorporating heterogeneity of sexual assault rates into the supplemental analysis presented here we are better able to emphasize uncertainty in the estimates of total sexual assaults derived using the at-risk population counts computed by ABSCIM. Secondly, we are able to underscore the model's utility as a policy analysis tool in a critical, yet data sparse issue space. Simply stated, in conflict settings we too often lack sufficient (or any) data on the prevalence of sexual assault, even in contexts like Ukraine where qualitative reporting indicates widespread abuse. In such situations there are three possible options to quantify the scale of this violence: assume such violence takes place but do not attempt to quantify it in real-time, perform retrospective analysis through surveys and interviews of at risk populations, and employ alternative strategies to quantify the scale of violence. Surveys and interviews offer the greatest path to precision, but come at the expense of time which is in limited supply during crises. In the main draft and here we attempt to demonstrate the third approach by using ABSCIM along with reliable external estimates of sexual assault rates to generate estimates of a critically important quantity (numbers of sexual assaults) which can also be updated daily as a conflict unfolds.

As noted in the main draft, estimates of wartime sexual assault exhibit significant variation and heterogeneity across conflict regions. To account for this, in the main draft we reported the lower-bound estimates for aggregate sexual assaults across all conflicts (14.9%) as well as for estimates using two conflicts which occurred in Europe: Bosnia and Herzegovina (37.6%) and Kosovo (3.4%). Here we extend this analysis in two ways by narrowing the temporal window within which we consider an agent to be in the at-risk population and by estimating these sexual assault figures using the full range of confidence estimates reported by Vu for the two European conflicts which may exhibit the greatest similarity to Ukraine.

Using ABSCIM we estimate the total number of female civilians displaced from regions within Ukraine which had a Russian military presence over staggered time intervals during the preceding week. We identify Russian military presence by subsetting areas within Ukraine which ACLED data indicate had at least one "Battle" producing a fatality indicating the presence of fighting Russian forces in the area. Unlike artillery strikes or other forms of remote violence, battles indicate the physical presence of military forces who may perpetuate acts of violence on civilian populations. We use four temporal windows to classify a civilians in proximity to Russian military ground forces as "at-risk": anytime during the preceding week (t to t-7), during the preceding five days (t to t-5), during the preceding three days (t to t-3) and during the same day only (t-0).

Table SB1 provides estimates of total number of possible female sexual assault victims using a range of temporal cutoffs for the presence of Russian military forces which range from presence anytime over the preceding week to a more restrictive cutoff that only considers at-risk female displaced on the day of the ACLED event (t-0). We use a maximum 7-day cutoff in order to align our main analysis and the assumed 7-day travel time employed by the ABM. Using the total estimated number of female Ukrainian refugees within proximity of Russian military forces we calculate the total possible number of sexual assault victims using the best *aggregate* estimates of wartime sexual assaults from Vu et al. [5] who estimate the percent of assault victims to be: 21.4% [14.9, 28.7]. Based on the computed values presented in Table SB1 we can see that the estimated 152.4 thousand assaults reported in the main text represents the conservative estimate using the full 7-day temporal window. However, by further restricting the "at-risk" population to include only those within proximity to Russian forces on the "day of" a battle we compute approximately 52 thousand assaults given the at-risk population identified by ABSCIM. However, these aggregate sexual assault rates incorporate many conflicts outside of a European regional context. Therefore, we further extend this analysis by leveraging estimates from two wars in Europe: Kosovo and Bosnia/Herzegovina.

Ukraine - estimated assaults

Assault rate	t to t-7	t to t-5	t to t-3	t-0
LB - 14.9%	152.4*	129.4	107.3	52.0
Mean - 21.4%	218.9	185.9	154.2	74.6
UB - 28.7%	293.6	249.3	206.8	100.1
ABSCIM Estimated at-risk population	1023.0	868.7	720.5	348.7

* indicates value reported in main text.
All estimates reflect values in thousands.

Table SB1: Aggregate rate

Table SB2 reports Ukraine sexual assault estimates using the at-risk population computed by ABSCIM within a 7-day window along with European conflict region-specific sexual assault rates reported in the supplemental appendix of Vu et al. [5] for Kosovo and Bosnia/Herzegovina. Using

the conservative lower-bound sexual assault rates for these two conflicts along with the ABSCIM computed at-risk population our analysis suggests between 34 thousand and 384 thousand assaults may have occurred during the initial stage of the war while large civilian populations were still present in Eastern Ukraine and attempting to escape encroaching military forces.

Ukraine - estimated assaults

Conflict estimated assault rate	t to t-7
LB - 3.4%	34.8⋆
Mean - 4.4%	45.0
UB - 5.7%	58.3

⋆ value reported in main text.
All estimates reflect values in thousands.

Kosovo rate

Ukraine - estimated assaults

Conflict estimated assault rate	t to t-7
LB - 37.6%	384.6⋆
Mean - 43.5%	445.0
UB - 49.5%	506.4

⋆ value reported in main text.
All estimates reflect values in thousands.

Bosnia rate

Table SB2: Regional-specific rates

This analysis of sexual assault rates during the initial phase of the Ukraine conflict employed demographically detailed and spatially precise estimates of at-risks populations within Ukraine generated by ABSCIM along with best estimates of sexual assault [5]. Using these two pieces of data our analysis here suggests with the most conservative assault rate (Kosovo) and at-risk population including individuals within proximity to Russian military forces within a 1-week period, approximately 34 thousand sexual assaults occurred during the initial stage of the Ukraine war. Given extensive qualitative reporting by news organizations and governmental organizations of the extensive nature of these assaults this conservative value likely understates the true scale of violence.

In this analysis we are not attempting to suggest that these ABSCIM derived sexual assault estimates are perfect. Rather we hope to demonstrate, given the demographically and spatially detailed output of ABSCIM, that we can compute this quantity using the best available information on sexual assault. Given extensive reporting early in the conflict on the widespread nature of these assaults as well as the need for humanitarian groups to have an informed idea on the scale of this form of violence this analysis demonstrates a useful and salient policy application for the model in crisis settings. Additionally, by highlighting the variation in wartime sexual assault we also draw attention to conclusions of other scholars: that wartime sexual assault prevalence requires further research, particularly for understanding factors that help to account for spatial variation in assault rates.

SB 2 Conflict model

We forecast discrete daily conflict events indexed by latitude and longitude by first fitting a Log-Cox Gaussian point process model with a spatial mesh. This modeling approach and spatial mesh allow us to identify a spatiotemporal random field defined by a stochastic partial differential equation that employs a Matern covariance function as a solution to estimate the continuous spatial dependence between conflict events over time within the field [1, 4]. Critically, the model allows us to estimate the location and timing of violence in a way that preserves the dynamics of the conflict apparent on the ground in Ukraine and therefore representative of violence in the country. We fit the model with INLA [2] using ACLED data of observed violent conflict events between February 24, 2022 through April 21, 2022 and then forecast violent events for an out-of-sample two-week period starting April 22, 2022 and running through May 05, 2022.

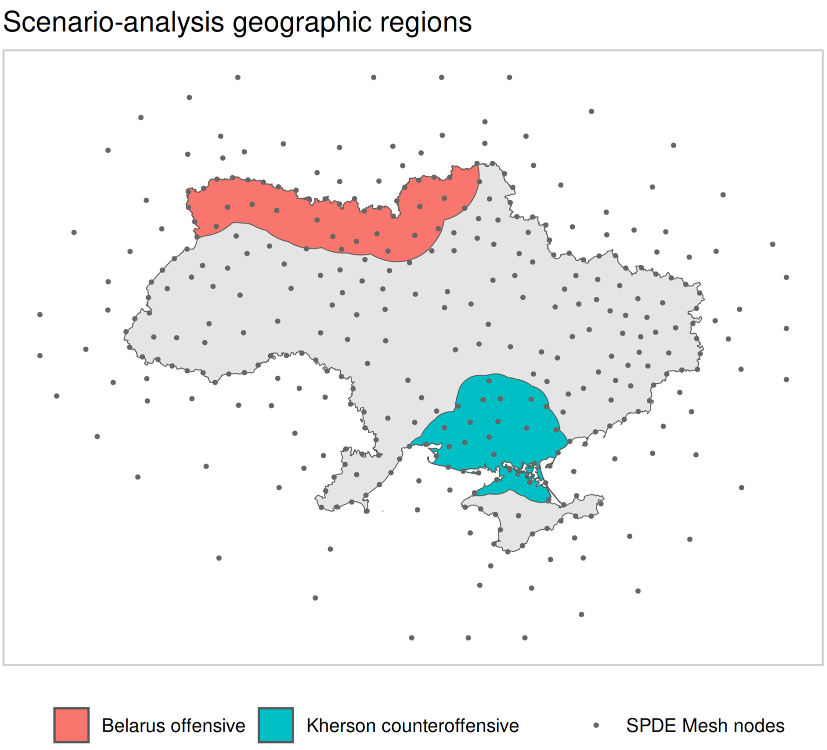

Figure SB1: Counterfactual scenarios - Geographic Scope

Using the estimates of the geostatistical model we forecast the spatial random field elements to produce geographically specific estimates of future daily conflict locations and intensity. By shocking the nodes of the spatial mesh from the model output we can simulate probabilistically informed estimates of conflict potential based on the persistence of past violence within a geographically defined location over time. Therefore, in contrast to descriptive empirical methods (such as spatial kernel density estimators), our estimates of conflict potential are constrained by the model to reflect real conflict processes likely to occur within Ukraine based on observed events and the trajectory of the conflict. Using estimates from the shocked spatial field forecasts, we then sample spatially indexed points based on the forecast expected daily number of predicted events in order to generate future conflict events which we then supply the ABM.

The conflict model assumes the following structural form:

$$\pi(Y|\lambda) = \beta_0 + X(s)\beta + \psi(s_i) \tag{1}$$

where $\pi(Y|\lambda)$ represents the point pattern of outcome Y conditional on the intensity surface λ indexed across continuous space, β_0 represents a structural intercept and $X(s)\beta$ represent covariates and slopes indexed at site specific (s) locations corresponding to observed events (if covariates are included in the model - they are not necessary), and $\psi(s)$ represents a Gaussian field approximated by a continuously indexed Gaussian random field. Specifically, $\psi(s)$ can be represented as:

$$\psi(s_i) \sim N(0, \Sigma) \tag{2}$$

where Σ represents the variance-covariance of the field as determined by the modeled spatial correlation function - here a Matern spatial decay function fit with an SPDE model:

$$\Sigma = \frac{\sigma^2}{\Gamma(\lambda)2^{\lambda-1}}(\kappa||s_i - s_j||^{\lambda} K_{\lambda}(\kappa||s_i - s_j||) \quad (3)$$

This modeling approach provides two crucial elements to forecasting conflict outcomes:

- a structural and spatially explicit component indexed at observed event locations and spatial mesh integration nodes captured by $\beta_0 + X(s)\beta$
- a spatially continuous Gaussian Markov random field (GMRF) captured by $\psi(s)$

Together these two elements can be used to forecast conflict trends as well as explore policy hypotheticals based on included covariates. Critically, the GMRF can be forecast independently of the structural component to gain leverage on the latent and spatially continuous potential for conflict across the fighting space. These estimates can then be used in conjunction with the structural component to forecast conflict hypotheticals from an empirically informed baseline.

To forecast plausible scenarios, we allow the adjustment to conflict shocks to occur gradually across space over a short time window. In producing these forecasts we allowed the conflict to respond to the shocks gradually over the course of a 5-day window in order to reflect shifting military material and personnel resources. Additionally, these scenario shocks were modeled in absolute terms across the fighting space to reflect fixed military resources. That is, an absolute gain in fighting in one location was compensated with reductions in fighting potential elsewhere. Fundamentally soldiers fight wars and this assumption incorporates the reality that there are a fixed number of soldiers in the Ukraine conflict. However, the approach we employ is flexible to facilitate counterfactual analysis of new conflict spaces opening in order to model refugee flows in such circumstances. Figure SB1 provides spatial domains of the two offensive scenarios.

SB 3 Comparison of ABM demographic distribution with IOM survey report

.

As an additional point of validation, we compare the IDP distribution of various demographic groups suggested by our model against the Round 1-Round 4 General Population survey conducted by the International Organization of Migration (IOM) [3] covering different periods within our time period of study. between March 24, 2022 and April 1, 2022.

As an example to provide more clarity, we look at Round 2 from Table SB3, the demographic distribution of male-female estimated by ABM is 45.35%-54.65%, which is quite similar to the survey report (41%-59%). Among the age groups reported in the survey, two distributions follow the model suggestion quite well. Finally, our model suggests that among the displaced households, around 10.15% households (5% in the survey) contain at least one infant (age $<$ 5) and 57.42% (57% in the survey) contain at least one elderly, following the reported distribution of these vulnerable groups quite closely. Such vulnerable households usually require medical and financial attention more than other households. The fact that our model can identify such households proves another pivotal utility from a humanitarian standpoint.

SB 4 Forecast Scenario Results

Figure SB2 shows the difference in migrant outflow from raions for the two conflict scenarios against the status quo scenario. As expected, raions with high deviation are mostly surrounding the spatial

Table SB3: Comparison between different demographic distribution from ABM with IOM survey reports.

Round	Group	Category	IOM Survey Report (%)	ABM Estimation (%) Median [lower bound, Upper bound]
1	Gender	Male	46	44.69 [43.1, 46.22]
		Female	54	55.31 [53.78, 56.9]
	Age	18-29	21	21.11 [21.07, 21.14]
		30-39	33	17.74 [17.65, 17.84]
		40-49	25	16.83 [16.69, 17.02]
		50+	22	44.32 [44.05, 44.53]
	Vulnerable	Infant	28	28.74 [24.83, 32.73]
		Elderly	56	56.56 [53.07, 60.25]
2	Gender	Male	41	45.35 [44.52, 46.16]
		Female	59	54.65 [53.84, 55.48]
	Age	18-29	19	21.62 [21.18, 22.13]
		30-39	32	17.9 [17.51, 18.14]
		40-49	19	16.71 [16.47, 16.9]
		50+	30	43.77 [42.87, 44.84]
	Vulnerable	Infant	5	10.15 [9.56, 10.81]
		Elderly	57	57.42 [52.54, 62.21]
3	Gender	Male	40	46.43 [45.72, 47.85]
		Female	60	53.57 [52.15, 54.28]
	Age	18-29	13	20.33 [18.89, 21.5]
		30-39	33	16.85 [15.78, 17.61]
		40-49	23	16.07 [14.81, 16.89]
		50+	32	45.79 (44.04, 50.52)
	Vulnerable	Infant	7	10.76 [9.61, 11.48]
		Elderly	57	58.35 [53.78, 62.99]
4	Gender	Male	37	42.72 [42.15, 51.14]
		Female	63	55.56 [48.86, 57.6]
	Age	18-29	15	19.61 [18.27, 20.28]
		30-39	30	16.75 [14.95, 17.66]
		40-49	22	15.93 [14.3, 16.49]
		50+	33	47.66 (45.64, 52.48)
	Vulnerable	Infant	7	10.7 [6.69, 12.26]
		Elderly	55	57.61 [56.25, 62.11]

domains of the specific scenario (northern part close to Belarus border for the Belarus Offensive scenario and southern part of Kherson for the Kherson Counteroffensive scenario.)

Figure SB3 shows the household depletion situation taking 6 Raions as samples. All these Raions are situated close to the Belarus border, making them likely candidates to be affected by the conflict scenario. The Raions in the top row observed significant differences in the Belarus offensive scenario (Around late April) compared to the offensive scenarios, whereas the ones in the bottom row behave similarly in both scenarios. Consequently, the number of remaining household agents for Raions in the bottom row by April 21 (the day before the conflict scenario begins) is close to or below 20%, denoting that more than 80% households have already left and likely that

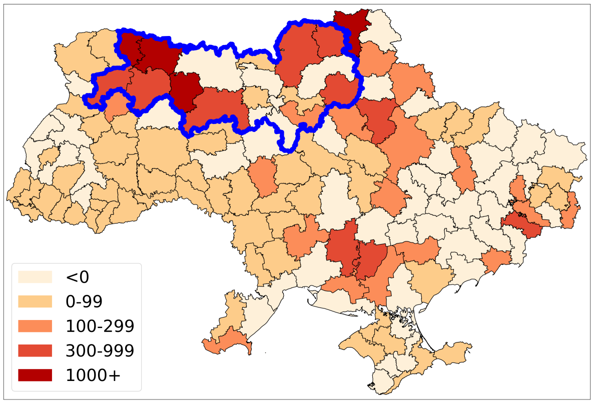

(a) Belarus offensive

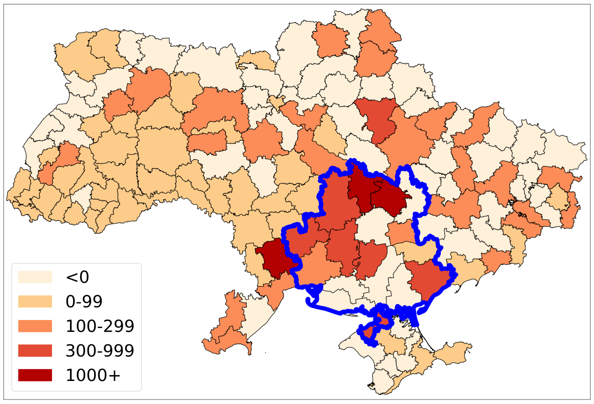

(b) Kherson counter-offensive

Figure SB2: Difference in migrant outflow with status quo for the two offensive forecast scenarios. Blue boundary surrounds the zone with a higher number of conflict events than status quo scenario.

the remaining households are not susceptible to migration either due to their location or their demography. However, the Raions in the top row have almost all the households remaining around April 21. Therefore, there are still enough households left to migrate should conflict events arise, causing the deviation in the outflow pattern in the offensive scenario.

References

[1] Elias Krainski et al. *Advanced Spatial Modeling with Stochastic Partial Differential Equations Using R and INLA*. Chapman and Hall/CRC, 0 edition, 12 2018. 3

[2] Finn Lindgren and Håvard Rue. Bayesian Spatial Modelling with *R* - **INLA**. *Journal of Statistical Software*, 63(19), 2015. 3

[3] International Organization of Migration. Ukraine — Internal Displacement Report — General Population Survey Round 2 (24 March — 1 April 2022). `https://dtm.iom.int/reports/ukraine-internal-displacement-report-general-population-survey-round-2-24-march-1-april`, 2022. [Online; accessed May 30, 2023]. 5

[4] D. Simpson, J. B. Illian, F. Lindgren, S. H. Sørbye, and H. Rue. Going off grid: computationally efficient inference for log-Gaussian Cox processes. *Biometrika*, 103(1), 03 2016. 3

[5] Alexander Vu et al. The Prevalence of Sexual Violence among Female Refugees in Complex Humanitarian Emergencies: A Systematic Review and Meta-analysis. *PLoS Currents*, 2014. 1, 2, 3

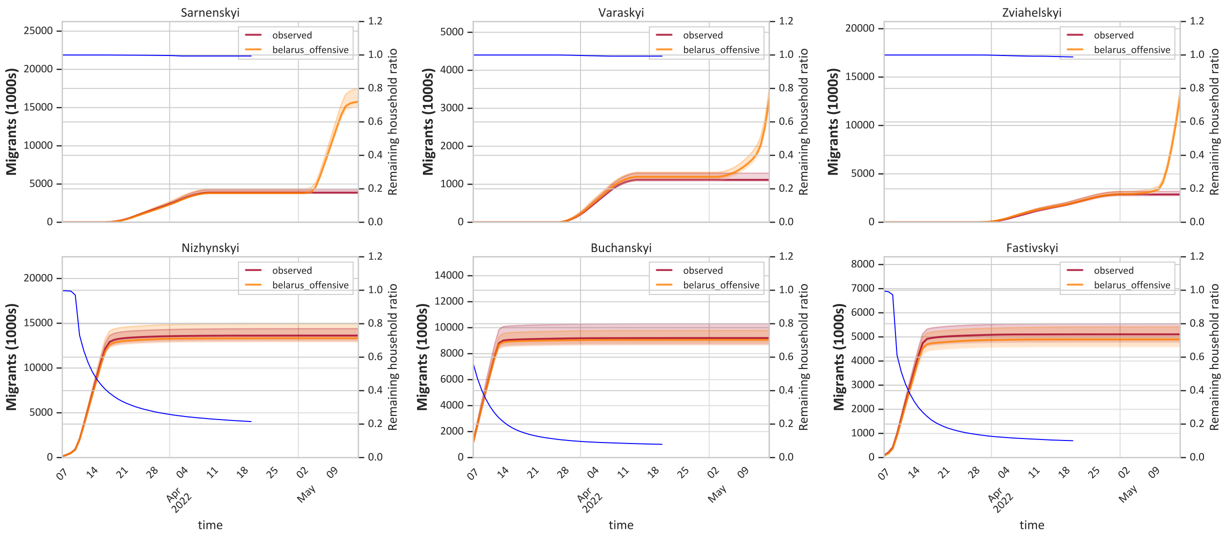

Figure SB3: Household depletion situation for sample Raions in Belarus Offensive scenario
